# The spiny relationship between parallel fibers, climbing fibers, and Purkinje cells

**DOI:** 10.3389/fphys.2025.1671271

**Published:** 2025-10-09

**Authors:** Stefano Masoli, Martina Francesca Rizza, Francesco Moccia, Egidio D’Angelo

**Affiliations:** ^1^ Department of Brain and Behavioral Sciences, University of Pavia, Pavia, Italy; ^2^ Department of Medicine and Health Sciences “V. Tiberio”, University of Molise, Campobasso, Italy; ^3^ Digital Neuroscience Center, IRCCS Mondino Foundation, Pavia, Italy

**Keywords:** Purkinje cell, cerebellum, spines, synapses, history, parallel fibers

## Abstract

Cerebellar Purkinje cells are one of the most complex neurons in the central nervous system and are well known for their extensive dendritic tree dotted by dendritic spines. PC spines receive excitatory synapses from parallel and climbing fibers and, although their morphological properties are comparable to those of other neuronal types, they show distinct extracellular and intracellular regulatory properties. Purkinje cell spine protrusion and helical patterning do not require nearby axons, as e.g., in pyramidal cells. Instead, Purkinje cell spines require structural proteins located on parallel and climbing fibers for their stabilisation and maintenance. The total spine number is influenced by scaffold proteins and eventually reflects the total dendritic length and local spine density. Purkinje cell spines were supposed to range up to over 10^5^ in rodents and 10^6^ in humans, but recent experimental data show that spines are less numerous than initially thought. Instead, they are endowed with mechanisms designed to improve their efficiency and differentiation. Some spines are double-headed, thereby enhancing Purkinje cell responses when the companion parallel fiber is stimulated. Other spines are single-headed and presumably endowed with slow neurotransmission mechanisms. Latest experimental data showed that glial cells modulate spines activity after a task or learning. Eventually, these multiple mechanisms can make each spine crucial in its own way for synaptic pattern recognition. In this review, we present the most recent advancements on Purkinje cell spines spanning their biochemical, structural, and functional properties, both in mice and humans, and propose a recalculation of the effective complement of spines and their activation by parallel fibers.

## Introduction

The first description of “protoplasmic processes,” currently called dendrites, was reported by Golgi in 1883, using his newly developed Golgi-method staining (Golgi, 1883; Bentivoglio et al., 2019). Five years later, the first image of cerebellar Purkinje cells (PC) spines was published by Santiago Ramón y Cajal but, until this seminal work, spines were discarded as staining artifacts (Ramón y Cajal, 1888; Yuste, 2015; Defelipe, 2025). Spines are small protrusions of a neuron cell membrane, canonically described with a mushroom-like shape, which can be observed on the dendrites and, sometimes, also on the soma (Lackey et al., 2024). These protrusions are usually elicited by a nearby axon and consolidated through synaptic activity, but there are exceptions to this rule. Spines are quite common in the central nervous system (CNS) and can be found on both excitatory neurons, such as pyramidal neurons (PN) (Davidson et al., 2020), and inhibitory neurons, such as the PC (Lee et al., 2004; Lee et al., 2005), striatal medium spiny neurons (Lanciego et al., 2012), and dopaminergic neurons (Jang et al., 2015). Their primary role is to create a confined space in which a tightly interconnected biochemical machinery can modulate the strength of the postsynaptic responses (Nimchinsky et al., 2002). Cerebellar PCs are inhibitory neurons most known for expressing a huge quantity of spines on their extensive dendritic tree. Their spines retain most of the properties common to other neuronal types, but they also exhibit critical differences. Spines in PN are primarily generated and consolidated through synaptic activity, while in PCs, internal and external control proteins are required. Moreover, the presence of 10^3^–10^5^ spines on PC dendrites brought about issues of how they are generated, maintained, consolidated, silenced, and raised hypotheses on mechanisms that might limit their extensive coding space (Marr, 1969; Albus, 1971). It was proposed that not all spines have a presynaptic partner, that some are silent or covered by glial processes, and that some others generate only slow responses mediated by metabotropic glutamate receptors 1 (mGluR1). Recent experiments showed that the number of spines is lower than previously thought, with 30–50 thousand synapses in mice and 300–500 thousand synapses in humans. However, several spines receive more than one parallel fiber (PF) and glial cells would not silence spines but make them more specific for certain activities. Therefore, PC spines are endowed with complex mechanisms that eventually fine-tune neurotransmission and synaptic plasticity. This review considers the most recent experimental advancements on PC spines and the plethora of proteins that are involved in balancing this delicate system.

## Cerebellum: Purkinje and granule cells

**FIGURE 1:**
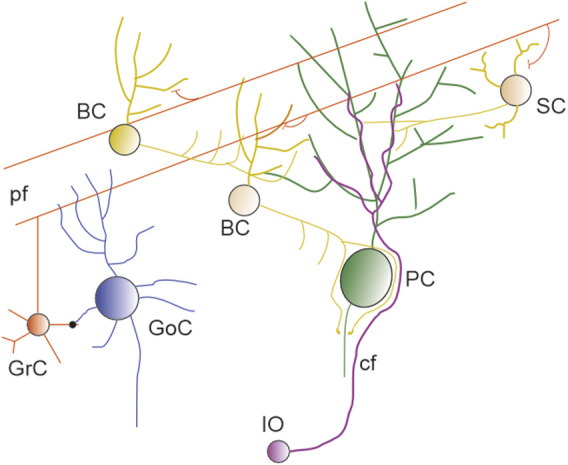
The cerebellar network. Schematics of the circuit showing GrC and PF (red), SC and BC (yellow and orange), PC (green), IO neuron and CF (violet), Golgi cells (blu).

The cerebellum is highly convoluted and, in humans, it can account for approximately 80% of the neocortex surface (Sereno et al., 2020). It is divided into 10 lobules, and each lobule is composed of 3 layers, termed granular (GL), molecular (ML) and Purkinje cell layer (PL). The GL contains the tightly packed granule cells (GrC) (Nguyen et al., 2023) and the sparser Golgi (GoC), Lugaro and Unipolar Brush cells, with the latter located primarily in the vestibular lobuli (lobuli IX and X). The PL contains the soma of PCs, the soma of Bergmann glia (BG) and the candelabrum cells (Osorno et al., 2022). The ML contains multiple types of inhibitory interneurons, the PFs, climbing fibers (CF) and the extensive dendritic arborisation of PCs (Figure 1). The ML is the location where the extensive territory occupied by PF synapses intermingles with the territory occupied by a few thousands of CF synapses.

Cerebellar GrCs, in conjunction with PCs, form the conserved primary input/output pathway of the cerebellar network (van der Heijden and Sillitoe, 2021). These neurons evolved millions of years before the first PN appeared in a nervous system and, during their long life, acquired characteristics that differentiate them from other neuronal cell types. One of these differences was recently highlighted by studying the amount of unmethylated DNA contained in their nuclei. This amount was unusually high for neurons, making them closer to glial cells (Tian et al., 2022; Tan et al., 2023).

### The Purkinje cell

PCs were described in 1837 by Johannes Evangelista Purkinje (Purkinje, 1837; Zárský, 2012; VoŽeh, 2015), who used the most advanced optical microscope available in the nineteenth century. Unfortunately, the limitations in the staining process restricted his study to the somatic region. Only in 1888, Santiago Ramón y Cajal, using an improved Golgi staining, described PC dendritic tree and spines (Golgi, 1883; Ramón y Cajal, 1888; Yuste, 2015).

It is one of the oldest neurons of the CNS and appeared in cartilaginous fishes 400 million years ago (Hibi et al., 2017; Mokhtar, 2020). These neurons are mostly known for their extensive and elaborated dendritic trees dotted by tens of thousands of spines. Even though not all species exhibit highly intricate dendritic trees (O’Brien and Unwin, 2006), they all have in common the presence of dendritic spines. This means that, over time, certain morphological properties were adapted by the evolution, while others were improved to support complex behaviour. Recent technical advancement showed that PCs are not a single family limited to just Zebrin- and Zebrin+ but there are multiple variants in zebrafish (Magnus et al., 2023) and up to eleven PC subtypes in mice (Khouri-Farah et al., 2025). In humans, the morphological evolution led to up to three distinct trees attached to the same soma (Busch and Hansel, 2023).

The importance of PCs as a computational powerhouse, in connection with the excitatory input transmitted from GrCs and the remainder of the neurons forming the cerebellar network, can be summarised by the wide range of abilities showed by the cerebellum. It is a motor coordination system (movement, balance) (Morton and Bastian, 2004), it is involved in higher cognition (Schmahmann, 2019), fear responses (Liu et al., 2010), language (Desmond and Fiez, 1998), emotion and sociality (Van Overwalle, 2024). Spinocerebellar ataxias (SCAs), which are progressive, degenerative, genetic diseases, are linked to multiple DNA mutations leading to various degrees of PC malfunctioning. In some cases mutations are so extreme to cause PC death through suppression of their intrinsic firing by hyperexcited ML interneurons (SCA1), by a reduction in Cav2.1 calcium (Ca^2+^) channel activity (SCA3), or a reduction of potassium (K^+^) channels currents (SCA6) (Hills et al., 2013; Hoxha et al., 2018; Huang and Verbeek, 2019; Egorova et al., 2023; Zhu et al., 2024). The cerebellar involvement in neurodegeneration was reported in Alzheimer’s disease (Mavroudis et al., 2019) and in Parkinsonism, where PC axonal dysfunction contributes to essential tremor (Schmahmann, 2019). Numerous physiological, biochemical and morphological studies of single cerebellar cells have been performed extensively on rodents, both in healthy and diseased conditions. The same approach can be used in humans only in very specific cases for ethical and technical reasons. This limitation can be mitigated by using non-invasive techniques that, although not providing the same quality data as single-cell recordings, can provide information useful to compare mice and humans. Unfortunately, as recently reported, the cerebellum is not taken into consideration in neurodegenerative disorders and is often neglected in multiple imaging studies (Wang et al., 2025).

### The granule cell, ascending axon and parallel fibers

Mice GrCs have a compact morphology with just three to six dendrites (Nguyen et al., 2023) and occasional branches (Houston et al., 2017). Some examples of the human GrC were reconstructed from *ex vivo* tissues, showing only three dendrites and a soma comparable in size with mice (Jacobs et al., 2014). The small dimension of the somato-dendritic sections makes them the most common neuron in the entire CNS (Tan et al., 2023). Their thin axon is split into an ascending axon (AA) followed by two PFs oriented in opposite directions (Palay and Chan-Palay, 1974b; Huang and Huang, 1998; Heck and Sultan, 2002). When an AA enters the ML, it can make synapses with multiple spines of the same PC, with AA/PF ratio in the order of 5%–10% (Lu et al., 2009). Instead, PFs can reach over 3 mm length in rats (Huang and Huang, 1998) and an average of 6.64 mm length in rhesus monkeys (Mugnaini, 1983). These fibers are canonically reported to make a single synaptic contact with a single spine of each PC they encounter. Recently it was shown that a single PF can make two or even more contacts with spines located in different locations of the same dendritic tree (Loschky et al., 2022). Even though PF intersect multiple PCs along their pathway, they only establish a stable connection with approximately half of them (Nguyen et al., 2023; Park et al., 2023).

There are few sources estimating the total number of PFs. An *in vivo* study using a sparse labelling method showed 540 PFs for a 200 × 200 μm^2^ section, accounting for just 0.38% of PFs in that section (Wilms and Häusser, 2015). The estimation for the total number of PF was about 142.000, which was in the same range as previously reported (Palay and Chan-Palay, 1974a). Another recent measurement performed on a 175 × 122 × 50 μm^3^ EM slab reported 33.900 (Park et al., 2023), which was suggested to account for 76.9% of the total number of PFs passing through the section. The total number of PF that can hypothetically pass through the section could reach 42.500 PFs. This variation in the number of PFs depends on the conformation of each lobulus and differences in the ML thickness between each sulcus, apex and the tissue in-between them. The changes in the ML thickness were studied in human sections showing that most lobuli have a thickness between 300 and 340 µm (Zheng et al., 2023). The smallest thickness was identified in lobule X (170 ± 80 µm), while lobules I and II showed the maximum thickness (360 ± 110 µm). In human sections of 200 μm × 160 μm, the estimated total number of PFs was 33.515 ± 36.261 (Kuo et al., 2011). Despite the large range of variation, the upper bound estimates are consistent with previous reports. More experiments will be required to elucidate the variability of PF number in each lobule, in both human and rodents as well as in health and disease.

## Purkinje cell spine properties

**FIGURE 2:**
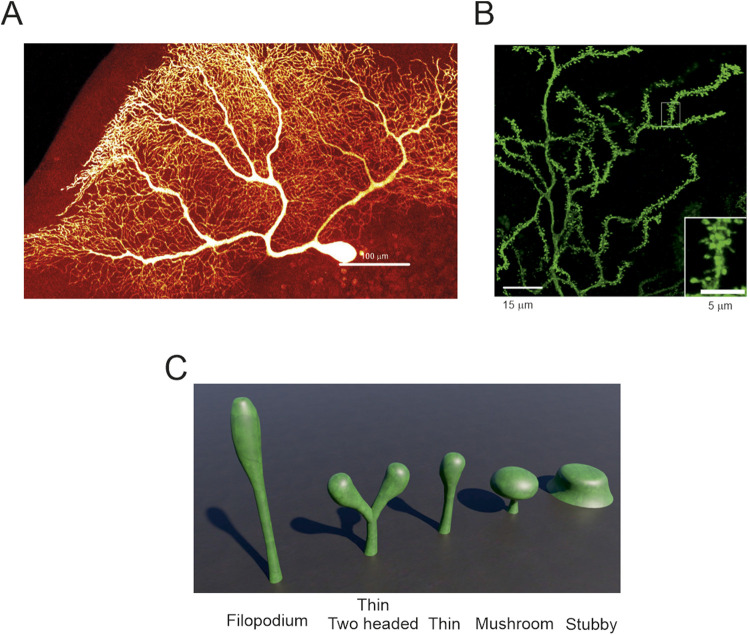
Purkinje cells spines – **(A)** Human PC reconstructed from post-mortem tissue (Masoli et al., 2024). At this resolution, spines are not evident. **(B)** Zoom-in of a portion the image in A showing spines decorating the dendrites. The inset further enlarges a dendrite crowded with many spines (modified from (Masoli et al., 2024)). **(C)** Typical spine shapes that can be found in PCs and in other neuronal types. The most common is the thin spine shape and, in PCs, 15% can have two heads (image built with Blender 3.6).

PCs are endowed with tens of thousands of spines that follow a helical pattern along the dendrites (O’Brien and Unwin, 2006; Parajuli et al., 2020) (Figures 2A,B). During mice development and until P20, spines are also expressed on the soma. They direct the CFs in the translocation process from the soma to the dendrites and *vice versa* for BC collaterals (Ichikawa et al., 2011). The requirement of having an intrinsic system to manage the spine distribution is a reflection of a profound difference with other neuronal types. PC spines follow the “Sotelo model,” which states that spines are intrinsically generated during the first and second week of development and a nearby axon is not required for their protrusion (Sotelo and Dusart, 2009; Dusart and Flamant, 2012; Verslegers et al., 2014). In contrast, PNs and other neuronal types follow the “Millers–Peters model,” which states that a spine can be protruded only when an axon and a dendrite are within a certain distance (Yuste and Bonhoeffer, 2004). It is a critical difference since a PC generates tens of thousands of PF - spine pairs with the ability to elaborate a near-infinite number of synaptic patterns. This supposed limitless in input/output could interfere with the encoding/decoding process in deep cerebellar nuclei (DCN), vestibular nuclei, and their transmission to the red nucleus and thalamus (Pugh and Raman, 2005; Gilbert and Rasmussen, 2025).

### One neck, not always a single head

The most stereotyped spine shape is called “mushroom” since it looks like a mushroom. It is canonically used to illustrate the morphological, biochemical and biophysical properties of a spine because there is a clear distinction between two adjacent regions called “neck” and “head” (Risher et al., 2014). The neck is generated by the outward bending of the cellular membrane followed by the head, which forms its terminal part. The mushroom-like shape has a neck and a head of similar length, but the head is significantly wider than the neck. When the difference between neck and head diameters is small, and the neck is multiple times longer than the head, the spine is called “thin”. If the difference in diameter and length between neck and head are non-existent, the spine is called “stubby”. When a spine does not show a clear separation between neck and head, it is called a “filopodium” (Li et al., 2023) (Figure 2C). A recent clustering analysis showed that spines should not be classified in predefined categories because they are a “continuum of shapes” with multiple intermediate forms (Pchitskaya and Bezprozvanny, 2020).

In rats, 75% of PC spines were described “thin” and only 25% were stubby, mushroom-like or with more than one head (branched) (Lee et al., 2004). A similar proportion was identified in mouse and human morphologies, with a higher number of thin spines compared to stubby, mushroom, and branched spines (Busch and Hansel, 2025). A recent technical advancement allowed to discern that 15% of spines in awake mice, and 7% in sleeping mice, have two heads on a single neck and in rarer cases, even three heads for a single neck (Loschky et al., 2022). Branched spines with similar features were also described in mouse and human PCs (Busch and Hansel, 2025) and in mouse hippocampal neurons (Mohrmann et al., 2024) suggesting a possible conserved property. Moreover, a rare “spine cluster” was uncovered in human morphologies, in which a single giant head showed multiple swellings acting as single spine heads (Busch and Hansel, 2025). Currently, it is not known if each head of the “spine cluster” contains an active synapse.

The other important part of the spine, the neck, can be wrongly classified by the low resolution of two photon microscopy and optical microscopes. This issue can increase the total number of stubby spines compared to the other known types (Tønnesen et al., 2014). In the majority of neuronal types, spines, necks and heads lay on the same plane but, in PCs, some heads can reach a 60° angle compared to the neck (Parajuli et al., 2020). Post Synaptic Densities (PSDs) are usually placed at the top centre of the postsynaptic membrane, but PC spines can angle their heads so the PSD can switch position and be placed even on the side of the head. With this flexibility, they can generate more occasions to find a nearby PF to establish a contact (Parajuli et al., 2020).

### Total surface area and dendritic length

In mammals, the width covered by PC dendritic trees passes from an average of 180 µm in P27 mice (Wilms and Häusser, 2015), to 300 µm in P90 Guinea pigs (Rapp et al., 1994) and to an average of 700 µm in 50–90 years old humans (Jacobs et al., 2014; Masoli et al., 2024; Busch and Hansel, 2025). The extensive dendritic tree in conjunction with rather large soma, averaging 20 µm in P27 mice and 35 µm in adult humans (Masoli et al., 2024), restricts the number of PCs to about 0.5% of all the neurons in the cerebellum (Tan et al., 2023). The total dendritic length, along with the linear spine density, is widely used to calculate the total spine number, which can vary among the cells. Since PCs are embedded in a 3D space, they do not occupy it entirely but are constrained by multiple parameters. The location in a lobule (apex or sulcus) (Nedelescu and Abdelhack, 2013) and the thickness of the ML (Liu et al., 2021; Zheng et al., 2023) dictate the overall shape of the dendrites and their extension. The same space contains other neurons (Stellate cells, Basket cells, candelabrum cells), fibers (PFs and CFs) and glial cells (Bergmann glia). Moreover, the cerebellar microvasculature contributes to the overall reduction of the space available for dendritic expansion. These factors can all limit the extension of their dendritic tree and the total number of spines. As summarized in Table 1, the total dendritic length can range from an average of 2,782.59 ± 671.12 µm in P27 mice (Masoli et al., 2024) to 7,900 µm in P63 mice (Gao et al., 2011; Takeo et al., 2021). In humans, it can range from 9,507 ± 1,053.13 µm (Mavroudis et al., 2021) to 63,645 ± 4,572 µm (Busch and Hansel, 2025). In all cases, the reported dendritic length may be underestimated due to the incomplete reconstruction of some thin dendrites. According to the data in Table 1, the variability reported above, and the type of technique used to reconstruct dendrites and spines (Li et al., 2023), the most common dendritic length ranges between 4,000 and 7,000 µm in mice and between 30,000 and 70,000 µm in humans. The human datasets are still very limited, and there are cases in which the total length amounts to only 10,000 μm, despite the use of good quality tissue sources and technique (Masoli et al., 2024).

**TABLE 1:** Total dendritic length and age. The table shows the age and the total dendritic length, which in some cases were estimated from graphs. The range is rather variable from 2 mm to 8 mm in mice. The variability is similar in human but on an order of magnitude more ranging from 9 mm to 67 mm.

Dendritic length (µm) (e = estimate from graphs)	Age	Paper
Mice		
2,782.59 ± 671.12	P27	Masoli et al. (2024)
4,430 ± 30	Various ages	Kumar et al. (2016)
6,004 ± 831	10–12 weeks	Busch and Hansel (2025)
7,000 (e)	P21	Liu et al. (2022)
2,500 (e)	P10	Takeo et al. (2021)
5,100 (e)	P14	
7,500 (e)	P21	
7,900 (e)	P63	
7,500 (e)	5–6 weeks	Gao et al. (2011)
Rats		
5,620.25 ± 2,504.09	P12-P21	Roth and Hausser (2001)
Humans		
10,500 (e)	Average 73	Mavroudis et al. (2019)
11,658.5 ± 5,734.2	Elderly	Louis et al. (2014)
(Vermis) 9,507 ± 1053.13	65.6 ± 6.0	Mavroudis et al. (2021)
(Hemispheres) 10,757.3 ± 1,666.24		
20,166.96 ± 15,248.58	50 and 90	Masoli et al. (2024)
63,645 ± 4,572	Various ages (>37)	Busch and Hansel (2025)

### Spine number per unit length

The number of spines for linear micron can vary between different studies, depending on the overall quality of the tissue and/or the techniques used. Usually, the number of spines is calculated from single dendritic branches using an optical microscope or from digitised images using confocal microscopes (Li et al., 2023). The stacked images, with the aid of specific software, can be reconstructed into a file and visualised with a computer to better study the distribution of spines in a 3D space (Gao et al., 2011; Busch and Hansel, 2025). The most cited estimates reported 4.5 spines/µm in feline PCs (Palkovits et al., 1971) and 17.2 spines/µm in rats (Napper and Harvey, 1988). While most reconstructions reported an average of 2 spines/µm, other studies reported 1.4 spines/µm (Gao et al., 2011) or up to 5.1 ± 0.61 (Busch and Hansel, 2025) or 7.1 ± 1.693 spines/µm (Parajuli et al., 2020). The spine number per unit length did not show associations with mice treatment, learning tasks or enriched environment, which are factors known to stimulate spinogenesis (Gelfo et al., 2016; Stevenson et al., 2021). Neither age nor animal strain appeared to exert an effect. Similar values were reported in human PCs, although with a limited number of investigations showing an average of 2 spines/µm. Recent human reconstructions showed an average of 6.9 ± 0.77 spines/μm (Busch and Hansel, 2025). The average distribution of spines, taken from various publications, is summarised in Table 2. The spine number per unit length obtained with most recent techniques and high-quality tissue can range between 4 and 8 spines/µm. In human, as previously discussed, the range was shown to range between 6 and 7 spines/µm (Busch and Hansel, 2025). It is not yet possible to define if 6 spines/μm is the lowest value since similar analyses were performed on high-quality tissue and yielded 2 spines/µm (Masoli et al., 2024). The human datasets do not yet cover the entire cerebellum, and regional differences can be a lot more critical compared to mice.

**TABLE 2:** Spines distribution in literature. The table contains experimental values collected in the literature about the average number of spines, the animal type and the age. Not all these data was available in the mentioned papers.

Number/micron linear	Type	Age	Paper
Mice			
1	C57/Bl6	Various	Kumar et al. (2016)
2	B6.Cg-Tg (Thy1-YFP)16Jrs/J	1-month old	Loschky et al. (2022)
2	Atoh1	P14	van der Heijden et al. (2021)
2	-	Adult	Peter et al. (2016)
2	C57BL/6N	4–6weeks	Toledo et al. (2019)
2	C57Bl6	(culture)	Campeau et al. (2013)
2	TLR4 knockout	4 months	Zhu et al. (2024)
2.2	C57BL/6N	6–12-18months	Hoxha et al. (2017)
2.7	Atxn2-CAG100-knock	9months	Arsović et al. (2020)
2–3	C57BL	3–10weeks	Sugawara et al. (2013)
3	Sv129 3 C57Bl/6	P78 to P204	Vecellio et al. (2000)
4.5 superficial	C57BL/6N Cas 9	P21	Liu et al. (2022)
5.5 deep			Liu et al. (2022)
5.1 ± 0.61	C57BL/6J	10–12 weeks	Busch and Hansel (2025)
7.10 ± 1.693	C57BL/6 male	12 weeks	Parajuli et al. (2020)
Rat			
1.1	-	5 weeks	Huang et al. (2012)
1.9			Huang et al. (2012)
2.07 ± 0.42 (Proximal)	Colture	-	Heintz et al. (2016)
2.93 ± 0.88 (Distal)			Heintz et al. (2016)
Camel			
1.2 and 2.2	-	-	Al-Hussain et al. (2022)
Human			
0.937 ± 0.93 (vermis)		65.6 ± 6.0 years old	Mavroudis et al. (2021)
0.98 ± 0.68 (Hemispheres)			Mavroudis et al. (2021)
1	-	Elderly	Louis et al. (2014)
1	-	Everage 73 years old	Mavroudis et al. (2019)
2	-	50 and 90 years old	Masoli et al. (2024)
6.81 ± 0.77 (Average) 5.37 ± 0.82 (Thin head) 0.49 ± 0.44 (large mushroom)	-	Various ages	Busch and Hansel (2025)

## Spine number estimates

### Early estimates of PC spines

One of the first estimates of the number of PC spines ranged between 80,000 and 100,000 spines in the feline cerebellum (Palkovits et al., 1971). This value was estimated considering 4.5 spines/µm, which matches current studies on rodents. However, the total dendritic tree length was not provided, making it difficult to assess the number of spines. Another estimate was performed on rat PCs and proposed 17.2 spines/µm. This value was multiplied by a total dendritic length of 9,941.5 µm, which was an average value obtained from a previous study (Palay and Chan-Palay, 1974a) yielding 175,000 spines per PC (Napper and Harvey, 1988). Compared to common spine estimation techniques, this approach was not based on the number of spines/µm but on an equation using spine volume densities as the main parameter (Napper and Harvey, 1988). This estimation, which is often used for reference, does not match the majority of experimental recordings and, even in the best cases, it is six-seven times larger than reality. Based on these numbers, many authors estimated that human PCs could reach up to one million spines or even more (Huang et al., 2014), but this value has recently been disproven (Busch and Hansel, 2025).

### Estimates based on the latest experimental data

As detailed in Table 1, the most common quantification of the spine density in mice and humans is 2 spines/µm. The average mouse dendritic length is approximately 5,000 μm, which, with 2 spines/µm, gives space to a maximum of 10,000 spines. Based on recent detailed morphological reconstructions, the spine number per unit length in mice can range up to 5.1 ± 0.61 (Busch and Hansel, 2025) or even 7.1 ± 1.693 (Parajuli et al., 2020) spines/µm. Even in these cases, with the same 5,000 µm dendritic length, the maximum number of spines increases up to a value ranging between 25,000 and 35,000 spines. Taking into consideration the longest recorded mouse dendritic tree (7,900 µm) (Takeo et al., 2021), and the highest number of spines/µm (7 ± 1.693) (Parajuli et al., 2020), the total number would reach 55,300 spines. Using the maximum spine density (6.81 ± 0.77 spines/µm) (Busch and Hansel, 2025) and the maximum dendritic length (63,645 ± 4,572 µm) (Busch and Hansel, 2025) reported in the human tissue, a similar calculation yields ∼470,000 spines, which is still only 47% of the proposed estimate of about one million. The estimates provided in Table 1 are primarily obtained from pieces of spiny dendrites and overlook differences in spine distribution due to regional variability or the absence of spines on main dendritic trunks. The most recent experimental data (Busch and Hansel, 2025) showed that human spines cover more dendritic length (95%) compared to mice (87%). This could mean that human PC have more spiny dendrites even in presence of multiple main trunks stemming from the soma.

### Fewer spines than expected but more critical than hypothesised

**FIGURE 3:**
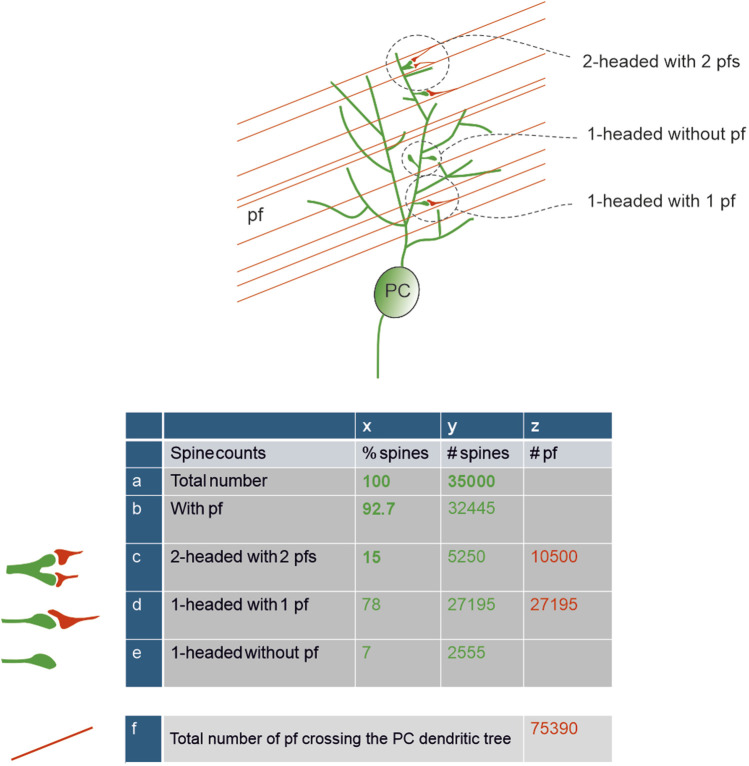
Total spine number and parallel fibers interaction – Schematics of PFs passing through the PC dendritic tree and of their connectivity with the PC spines. The insets illustrate the three main types of spine contacts. The table shows calculations of the number of spines (green) and PFs (red). Numbers in bold are derived from literature. The total number of spines, 35000, was taken from (Liu et al., 2022), the percentage of spines contacted by PFs and of double-headed spines was taken from (Loschky et al., 2022). The number of PFs crossing the PC dendritic tree is calculated assuming a contact rate of 50% (Park et al., 2023).

Based on the information given above, the total spine number is lower than the most cited estimations, but this reduction may not be a negative factor after all. The question turns into how synaptic integration over fewer spines can generate an effective response able to modulate DCN and the vestibular nucleus (Gilbert and Rasmussen, 2025). Due to the initial estimate of a very high number of spines, one argument used to reduce their total number was that ∼90% of them had no presynaptic partner, i.e., they were silent (Isope and Barbour, 2002). This hypothesis has recently been challenged by the discovery that 92.7% of spines do present a synapse (Loschky et al., 2022). Thus, the number of spines is lower than initially thought, but the number of spines featuring a synaptic connection is significantly higher. This evidence is in accordance with recent estimates leveraging advanced recording techniques to show that, in mice, there are up to 42,000 PFs (Park et al., 2023), which would yield about 40,000 synaptic pairs (PF–spine) if 93% of them synapsed with a PC. In close agreement, a recent estimate of spine density and total dendritic length (Liu et al., 2022) allowed to calculate the number of ∼35,000 spines, which we will use for all subsequent calculations (Figure 3).

A critical point is that not all the connected spines appear to have the same functional properties. Approximately 15.1% ± 3.6% of all spines are double-headed, receive one PF per head, and have been suggested to be more “eloquent” compared to the typical single headed spine (Loschky et al., 2022). The response of two or more PFs on the same spine can be elicited by the synchronous activation of GrCs, increasing their overall postsynaptic current and potentially resulting in a somatic response. Moreover, 16% of single-headed spines, which express mGluR1 mediated slow responses, may be critical in the overall synaptic pattern recognition. By combining this observation, 7% of synapses would remain orphan and therefore fully silent, 15% double-headed and fast-responding, and 78% single-headed, either slow-responding (16%) or partially silenced by glial cells (see below). This picture yields an estimate of up to a maximum of 85% putative silent synapses, approaching earlier estimates of 90% (Isope and Barbour, 2002; Brunel et al., 2004), but redefining their nature to include, in addition to null responses, also slow and partial responses.

As a special morphological feature of PCs, each spine is surrounded by BG, which forms multiple types of peri-synaptic astrocytic processes (PAPs) (Tao-Cheng, 2025). This affects the computation of active spines since glial cells can remodel spine structure by nibbling pieces of the spine membrane, a process that was reported to modulate their activity after learning (Morizawa et al., 2022). This is in contrast to another hypothesis, claiming that glia covered spines to make them unresponsive (Lippman Bell et al., 2010).

In aggregate, the count of active spines on the PC dendritic tree is complicated not just by the anatomical connectivity but also by specific processes that can regulate their effectiveness. To summarise, even though the average number of spines is probably ∼35,000 in mice and ∼360,000 in humans, 93% are connected to a presynaptic partner, 15% (double-headed) have high efficiency, while 78% (single-headed) have low efficiency (either modulated by glial PAPs or generating slow metabotropic responses). Similarly, the number of PFs effectively conveying information to a PC is also difficult to establish. It has been reported that PCs make synapses only with about half of the PFs traversing their dendrites (Nguyen et al., 2023; Park et al., 2023), amounting to ∼76,000 PFs crossing the PC dendritic tree, with ∼27,000 synapsing on double-headed spines and ∼10,000 synapsing on single-headed synapses (Figure 3).

## Molecular properties of Purkinje cell spines

Even thou the total spine number is lower than the original estimates, it does not detract from the fact that each spine is covered and contains multiple protein types and enzymes. These can be broadly subdivided into: a) structural proteins, which are fundamental to preserve the connection with the presynaptic side (i.e., PF and CF) through a series of transmembrane and secreted proteins; b) ionic channels and synaptic receptors, which allow the generation of action potentials and local increases in the intracellular Ca^2+^ concentration; and c) structural proteins and enzymes involved in the control of synaptic plasticity, Ca^2+^ buffering and receptors turnover.

### Structural proteins between spines and parallel fibers

The tripartite complex that stabilises PF-PC spines require: a) the GluD2 receptor on the spine surface, b) the neuropeptide cerebellin (Cbln1), and c) the presynaptic cell adhesion protein neurexin (Nrxn) on the PF membrane (Paul et al., 2024).

#### The GluD2 receptor

A critical structural protein is the GluD2 receptor (Kakegawa et al., 2009; Burada et al., 2022), whose structure was recently reconstructed with cryo-EM microscopy (Burada et al., 2020). GluD2 is a member of the glutamate receptor (iGluR) family encoded by the *GRID2* gene and has long been regarded as an “orphan” receptor, as it is not gated by glutamate (Naur et al., 2007; Yuzaki and Aricescu, 2017; Brunetti et al., 2024). It can act as an ionotropic receptor only in the Lurcher mutation (p.Ala654Thr), in which the protein quaternary structure is twisted in a constitutively open state (Selimi et al., 2003). This abnormal open state can be closed by D-serine or Glycine (Itoh and Yuzaki, 2024) and enhanced by extracellular Ca^2+^ (Hansen et al., 2009). In both cases, the alteration in GluD2 activity can push PCs into a hyper-excited state that ultimately leads to cell death in about two post-natal weeks. When the cerebellum is still immature, D-serine released by BG is critical to generate long-term depression (LTD) because GluD2 regulates the trafficking of AMPA (α-amino-3-hydroxy-5-methyl-4-isoxazole propionate) receptors (Kakegawa et al., 2011). The receptor is primarily expressed in cerebellar PCs (Itoh and Yuzaki, 2024), while it is less abundant in cerebral and hippocampal neurons (Konno et al., 2014). In all cases, GluD2 promotes synaptogenesis (Khan, 2017), thereby increasing the number of spines (Spanaki et al., 2024). GluD2 stabilises spines and promotes postsynaptic LTD both in the immature and mature cerebellum (Kakegawa et al., 2011). Deletion of GRID2 causes ataxia in humans (Hills et al., 2013) and can be rescued in mouse cultures by injections of GluD1 in the PC soma (Ryu et al., 2012). Another critical activity performed by GluD2 is the separation of territories occupied by PF synapses and CF synapses. Its absence causes the aberrant development of CF collaterals and synapses in the PF territory (Ichikawa et al., 2016).

The adaptor protein complex 2 (AP-2) and Glutamate Receptor Delta 2 Interacting Protein 1 (GRID2IP) were recently found to be critical for the balance of PF-CF territories. Loss of the two AP-2 isogenes, i.e., Ap2a1 and Ap2a2, in PCs causes the degradation of GRID2IP and an increased expression of GluD2. This reduces the CF-PC territory and increases the PF-PC territory, making PCs more excitable. This leads to morphological degeneration, early PC death and Spino Cerebellar Ataxia type 1 (SCA1) (Tolve et al., 2025).

#### Cerebellin, an adaptor protein

There are four variants of the secreted protein Cbln, which are encoded by four genes (*Cbln1-4*) (Südhof, 2023). They can interact with both GluD1 and GluD2 and with the various Nrxn isoforms only if these contain an insert in the alternatively spliced sequence 4 (SS4) (Uemura et al., 2010). The first Cbln, as suggested by the name, was discovered in the cerebellum: it is secreted by cerebellar GrCs and interacts with Nrxn to form the tripartite complex that stabilizes PF-PC synapses. Cbln1 expression is quite low at birth, but it undergoes a 20-fold increase and thereby becomes the most expressed cerebellar isoform during the postnatal development. Conversely, Cbln2 is highly expressed before birth but is strongly downregulated during the postnatal phase and reaches a very low expression level in adulthood (Seigneur and Südhof, 2017). Cbln4 has a slim expression in the cerebellum, but it was recently shown that it is the first Cbln isoform downregulated in SCA2 (Arsović et al., 2020) followed by Cbln3, which is highly critical for the maintenance of PF-PC synapses. Cbln3 cannot be secreted from PFs unless it associates with Cbln1 and, when it reaches the synaptic cleft, accumulates and modulates Cbln1 activity. This activity was explored in mice with a KO for Cbln3, which increased sevenfold the expression of Cbln1. Instead, the KO of Cbln1 completely eliminated Cbln3 from the synaptic cleft (Bao et al., 2006; Iijima et al., 2007; Larsen, 2021). Cbln1 is also critical for downregulating the formation of inhibitory synapses, mainly from SCs, on the PC dendritic tree (Ito-Ishida et al., 2014b) (see the inhibitory section). It has recently been discovered that Cbln1 and GluD2 are not only critical for maintaining spines and their presynaptic partners, but even for the dendritic tree development. A total KO of GluD2 does not influence the shape of PC trees, but a sparse KO or an increase in Clbn1 and GluD2 proteins disrupts the morphological properties of PCs (Takeo et al., 2021).

#### Neurexin, type I cell adhesion protein

Three isoforms of Nrxn are encoded by three genes (*Nrxn1-3*) but they are highly rearranged through alternative splicing (Fuccillo et al., 2015; Südhof, 2017). Nrxns are so important for the survival of GrCs that a KO of these proteins is fatal even in cell cultures. This condition was reversed by the application of brain derived nerve factor (BDNF) and partially rescued by insulin-like growth factor-1 (IGF-1) (Uemura et al., 2022). BDNF is a neurotrophic factor that is postulated to have an autocrine or paracrine activity on GrCs axons. This substance is released by GrCs axon under control of Nrxns. They are critical for the creation of the presynaptic machinery, which is activity-induced through action potential-dependent Ca^2+^ entry. Multiple combinations of *Nrxn* KO showed that the different isoforms are interchangeable since the cerebellum shows no structural defects when only two out of three Nrxn isoforms (Nrxn1/2, Nrxn2/3 and Nrxn1/3) are genetically deleted (Uemura et al., 2022). Nrxn2 was found to be critically involved with Cbln1 in the regulation of GrCs axonal guidance and growth. These proteins act as cues during development and elongation of the axon in an autocrine manner (Han et al., 2022) Figure 4A.

**FIGURE 4:**
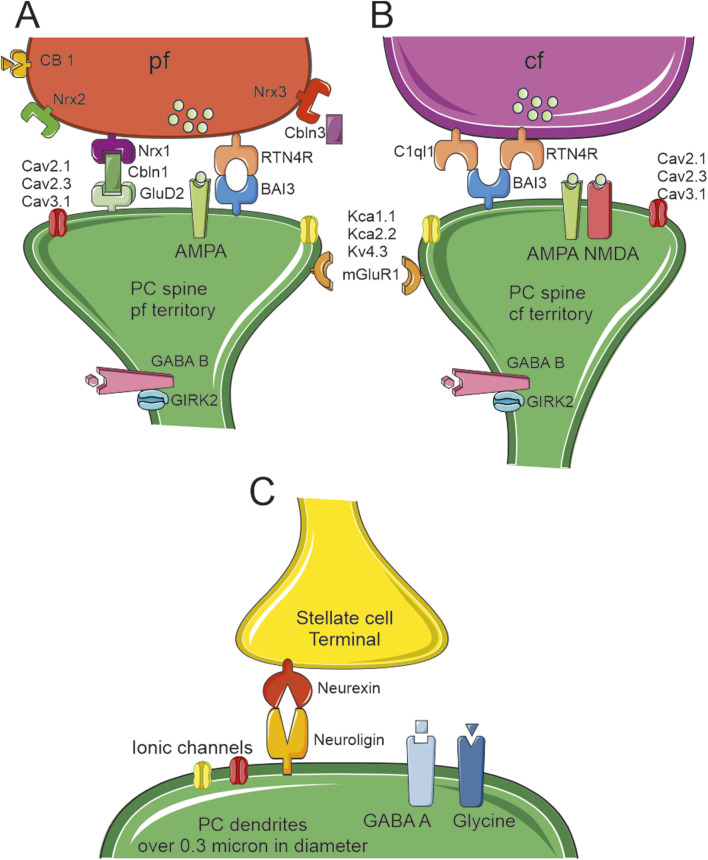
Proteins linking the pre and post synaptic sides. Schematic drawing of the main molecular components of PC spine synapses. **(A)**The PF terminal expresses three types of neurexin (nrx1-2–3) with nrx1 linked to the postsynaptic GluD2 through cerebellin (Cbln3). This is the main system that keeps PF and PC spines connected together. A second system, RTN4R and BAI3, can be found in some PF–PC synapses (although this complex is more typically expressed in CF–PC synapses). These spines expressed AMPA receptors, three types of Ca^2+^channels (Cav2.1, Cav2.3, Cav3.1), and three types of K^+^channels (KCa1.1, KCa2.2 and Kv4.3). The GABAB receptor is expressed on the spine neck along with GIRK2. **(B)**The CF terminal expresses RTN4R and C1ql1 linked to the postsynaptic BAI3. This is the main system that keeps CF and PC spines connected together. The CF spines express both AMPA and NMDA receptors, and (possibly) the same three types of Ca^2+^and K^+^channels as the PF spines. As in PF spines, CF spines express the GABA B receptor and GIRK2 on the spine neck. **(C)**The stellate cell synaptic terminals end on PCs dendrites and they are kept in place by presynaptic Neurexin and postsynaptic Neuroligin. The postsynaptic side hosts GABAA and Glycine receptors. PCs dendrites have multiple types of ionic channels that can change depending on their diameters [for reference supplemental materials (Masoli et al., 2024)].

#### Scaffold proteins between spines and climbing fibers

CFs are the terminal part of axons belonging to neurons located in the inferior olive nucleus. These fibers are critical to deliver the graded control correction signals capable of changing PC activity through the activation of PF-PC synaptic plasticity (Hansel et al., 2001; Coesmans et al., 2004; Jörntell and Hansel, 2006; Hoxha et al., 2016; Boele et al., 2018). The majority of PCs in mice shows a single trunk stemming from the soma, but PCs with two trunks stemming from the soma can be found in lobuli IX and X (Nedelescu et al., 2018). This is even more evident in human reconstructions, where three distinct branches were frequently observed (Busch and Hansel, 2023; 2025; Masoli et al., 2024). Similar to the previously defined tripartite complex, a complex of two proteins is required to stabilise a CF on PC spines: a) the secreted C1ql1 complement family protein and b) the brain angiogenesis inhibitor 3 (BAI3/ADGRB3) protein, which is an orphan receptor of the adhesion G-protein-coupled receptors (GPCR) (Sigoillot et al., 2015). Both GluD2 and BAI3 can be found in the immature PCs when the PF and CF synaptic territories are not yet defined. After the stabilisation of the synaptic territories, some spines belonging to the PF territory keep BAI3 on their membrane surface and connect RTN4R located on the PF presynaptic side (Paul et al., 2024). The presence of a single winner CF has been proved wrong for humans (Busch and Hansel, 2023) since there can be more than one depending on the number of main trunks. In some cases, more than one CF has been observed in rodents too (Nishiyama and Linden, 2004; Piochon et al., 2014). To guide the rodent CFs shift, transient somatic spines are generated lasting only until P20 and used by both CF and BC axon collaterals (Ichikawa et al., 2011). After P21, only the winner CF can be found on dendritic spines, showing a larger volume, extensive PSD and more AMPA receptors compared to somatic spines and dendritic spines of the looser CF. This process is coordinated by the Rab3-interacting molecule RIM, which can also be found in PFs (Nitta et al., 2025). Progranulin release by PCs acts as a retrograde signal activating sort1, which increases the release probability of the presynaptic terminals of the CF that have translocated from the soma to the dendrites (Uesaka et al., 2018). Dysregulation of either C1ql1 or BAI3 in the adult allows the formation of new synaptic contacts between nearby CF branches and the upper part of PC dendritic tree (Aimi et al., 2023). The KO of either Cbln1 or C1ql1 causes the disruption of at least 50% of PF and CF synapses (Paul et al., 2024). As observed upon the KO of GluD2, the absence of Cbln1 disrupts the CF territory, increasing its presence in territories normally occupied by PF synapses Figure 4B.

#### How structural proteins control inhibitory synapses

Early work described that BC collaterals interact with PC somatic spines for 2 weeks until P20 (Ichikawa et al., 2011). After this point in the development, no other evidence of inhibitory interneurons synapsing with PC spines was provided, not even SCs, which make synapses directly with dendrites. This is different compared with the morphological reconstructions and electrophysiological recordings performed in PNs, in which some spines are dedicated to receiving inhibitory synapses (Boivin and Nedivi, 2018). The absence of SC synapses on PC spines is due to the same Cbln1 that controls the PF territory. The number of SC and CF synapses was significantly increased in cerebellar slices from Clbn1/DluD2-deficient mice (Ito-Ishida et al., 2014a). This investigation further showed that the lack of Cbln1 also increased the density of the Vesicular Gaba Transporter (VGAT)-positive puncta, which are a marker of GABA- and glycine-containing inhibitory terminals (although with some regional differences in P11 mice). Since Cbln1-GluD2 signalling can control the territory occupied by VGAT, this finding confirms that the PF-PC synapses can hetero-synaptically control the generation and stabilisation of molecular layer interneurons (MLI)-PC synapses (Ito-Ishida et al., 2014a). Cbln1 finely regulates synaptogenesis through the Src-family protein tyrosine kinase (SFK) pathway (Ito-Ishida et al., 2014b). PC-expressed Neuroligin can interact with Nrxn expressed by MLI to generate the complex that stabilises these synapses (Südhof, 2008; Zhang et al., 2015; Park et al., 2018). The absence of Neuroligin or Nrxn can impair mature synapses but is not involved in synaptogenesis, which has been recently attributed to Dystroglycan. SC synapses cannot be found on spines since they do not co-localise with markers of excitatory synapses (Jahncke et al., 2025). Global KO of Neuroligin 2 reduced the inhibitory input from MLIs to PCs and suppressed pruning of CF synapses (Suk et al., 2025) (Figure 4C).

### Ionic channels and receptors in PC spines

The majority of synaptic receptors, ionic channels and internal biochemical pathways which control the postsynaptic plasticity are within the conglomerate of scaffolding proteins forming the PSD (Harris and Stevens, 1988; Cramer and Gao, 2013; Chen et al., 2022).

#### Ionic channels

PCs are endowed with multiple voltage-dependent ionic channels distributed over the different cell compartments (Masoli et al., 2015). Some channels have an axosomatic expression, but the majority are somato-dendritic. Some have a higher expression on the proximal part of dendritic trees, whereas others cover the entire tree, including dendritic spines. The most known ionic channel, the P-type high voltage-activated (HVA) Ca^2+^ channel (Cav2.1) can be found everywhere and can act alone or cluster with big (BK, KCa1.1) and small conductance Ca^2+^-dependent K^+^ channels (SK2, KCa2.2) (Indriati et al., 2013; Luján et al., 2018a). The modulation of the spike amplitude is under control of A-type K^+^ channel (Kv4.3) and the rebound excitation from negative potential is modulated by a low voltage-activated (LVA) Ca^2+^ channel (Cav3.1) (Otsu et al., 2014; Alfaro-Ruíz et al., 2020). The R-type HVA Ca^2+^ channel (Cav2.3) is found in spines, but is not critical in controlling the overall PC electrical responses (Otsu et al., 2014).

The physical length and the absence of voltage-dependent sodium or Ca^2+^ channels from the necks (Araya et al., 2006) can generate a local filtering system. The G-protein inward-rectifier K^+^ channels 2 (GIRK2), expressed on both necks and heads (Luján et al., 2018b), could also act as a modulatory system. It can shunt the forward propagation of weak signals from the spine to the rest of the dendrite and, at the same, filter the back propagating spikes from the axosomatic compartments by promoting membrane hyperpolarization.

#### Ionotropic receptors (AMPA, NMDA)

The majority of neurons express both AMPA and NMDA receptors on their spines, whereas PCs express a majority of spines with only AMPA receptors. The absence of postsynaptic NMDA receptors can be due their presynaptic expression on PF (Schonewille et al., 2021). Postsynaptic NMDA receptors are instead expressed by spines belonging to the CF territory (Piochon et al., 2007; 2010). The AMPA receptor subtype expressed by human PC spines comprises all the known subunits (GluR1 – GluR4) in their flip and flop splice variants (Tomiyama et al., 1999). The highest expression was reported for GluR1 (Castejón and Dailey, 2009), GluR2 (Liu et al., 2010) and GluR3 (Loschky et al., 2022). AMPA receptors in PCs are almost impermeable to Ca^2+^ ions, since they contain the GluR2 subunit. This subunit is critical for the AMPA assembly since it controls receptor kinetics, conductance of single-channel, and Ca^2+^ permeability. The passage of Ca^2+^ is limited by the presence of an arginine residue at position 607 (R607) that introduces an additional positive charge in the pore (Isaac et al., 2007). According to recent experiments, Ca^2+^ permeability can be modulated by the transmembrane AMPAR regulatory protein (TARP) and cornichon auxiliary subunits, modifying the known properties of GluR2 subunits (Miguez-Cabello et al., 2025). The paired pulse facilitation of AMPA receptors differ depending on the source of the presynaptic innervation: AMPA receptors expressed by spines belonging to the PF territory present a strong facilitation (Schmidt, 2019), whereas those expressed in the CF territory showed a strong depression after a single pulse (Zhang et al., 2020). This difference has been proven, not only with electrophysiological approaches but also by assessing the expression of the glutamate transporters Vglut1 and Vglut2. The former is associated with the majority of PFs and the latter only with CFs (Mao et al., 2022). These AMPA receptors are assisted primarily by NMDA GluN2A subunits and, to a lesser extent, by GluN2B subunits (Renzi and Cull-Candy, 2007). GluN2A has a high opening probability that facilitates Ca^2+^ entry, whereas GluN2B has half the value of GluN2A opening probability but shows longer openings (Santucci and Raghavachari, 2008). These receptors, along with Cav2.1 Ca^2+^ channels, play a critical role in Ca^2+^-dependent facilitation and depression (Kim et al., 2008; Benton and Raman, 2009; Adams et al., 2010).

PCs synthesise and release glutamate from their dendrites until the fourth postnatal week (Crépe et al., 2011). This autocrine activity on spine receptors is useful for depolarisation-induced suppression of excitation (DSE), and for depolarisation-induced potentiation of inhibition (DPI) (Crépe et al., 2011). Glial cells are usually in charge of clearing the excessive glutamate from the cleft, and this is one of the multiple activities performed by BG, which expresses the glutamate transporter EAAT2. Contrary to other neuronal types, PCs express the EAAT4 transporter in the spine perisynaptic region (Dehnes et al., 1998; Tao-Cheng, 2025). In postischemic mice, the low expression of this transporter causes excitotoxicity and cell death (Yamashita et al., 2006).

Three members of the ionotropic P2X receptors (P2X_2_, P2X_4_, and P2X_6_), which are non-selective cation channels gated by ATP, have been detected on spines belonging only to PF territory (Rubio and Soto, 2001). These ionotropic receptors mediate both membrane depolarization and Ca^2+^ influx in some regions of the CNS (Mut-Arbona and Sperlágh, 2023), but their physiological role in cerebellar PCs is yet to be determined.

#### Metabotropic receptors (GABAB, mGluR1)

Compared to PNs, PCs do not have spines capable of receiving inhibitory inputs from GABAergic interneurons. However, spines belonging to both PF and CF territories express extra synaptic GABAB receptors, which can cluster with GIRK2 channels on spine necks and with Cav2.1. The activation of GABAB receptors enhances the depression of the synaptic currents (AMPA-mediated fast synaptic currents and mGluR-mediated slow synaptic currents) induced by glutamate in spine heads (Tabata and Kano, 2006) and is responsible for PC hyperpolarization (Luján et al., 2018b). The presence of GABAB receptors, GIRK2 ionic channels, and the correlation between neck lengths and electrical activity (Araya et al., 2006) could generate a filtering property that could reduce the noise to signal ratio of each spine.

Another major player in generating slow responses is mGluR1. The CF that wins the competition and becomes stabilised on PC specific main trunk is under control of mGluR1 located on spines, AMPA receptors located on PFs and NMDA receptors located on MLI (Nakayama et al., 2024). The cannabinoid receptor CB1 is located on the presynaptic PF (Buceta et al., 2020) and is stimulated by mGluR1 through a signalling cascade that generates 2-arachidonoylglycerol (2-AG) and anandamide as retrograde messengers (Marcaggi, 2015; Hoxha et al., 2016). This pathway is critical because it reduces the release probability of PF in a Ca^2+^- and glutamate-dependent manner (Safo et al., 2006). Accordingly, mGluR1 stimulates phospholipase Cβ (PLCβ) to cleave phosphatidylinositol-4,5-bisphosphate (PIP_2_) into inositol-1,4,5-trisphosphate (IP_3_) and diacylglycerol (DAG) (Negri et al., 2020). DAG is hydrolyzed into 2-AG by DAG lipase and can thus serve as a retrograde messenger to reduce glutamate release from PFs (Safo et al., 2006). While DAG promotes the inhibitory inputs at the PF-PC synapse, the other branch of the signalling cascade, i.e., IP_3_, maintains the presynaptic function by inducing the secretion of BDNF, which acts as a retrograde messenger to increase the glutamate release probability (Furutani et al., 2006). The chronic suppression of mGluR1 and IP_3_ profoundly reduce the release probability. A similar activity can be induced by applications of BDNF (Furutani et al., 2006). The weight of DAG vs. IP_3_ signalling at the PF-PC synapse could depend on their different rates of degradation upon PLCβ activation (Raghu et al., 2019; Joensuu et al., 2020).

### Inside a PC spine

The intracellular molecular mechanisms of PC spines are highly specialized and include several enzymatic cascades, molecular motors, and a specialization of the endoplasmic reticulum (ER) called spine apparatus. These are instrumental in ensuring spine neurotransmission, plasticity, and motility.

#### Cytoplasmic molecules and the spine apparatus

The ER in PC dendritic spines is central to Ca^2+^ dynamics and synaptic plasticity. It regulates intracellular Ca^2+^ homeostasis, which is essential for synaptic function and plasticity. The ER network extends into the dendrites and spines with specialised sub domains, such as spine-associated ER and smooth ER tubules, contribute to localised Ca^2+^ dynamics. The ER serves as a major Ca^2+^ store, modulating LTD and other forms of synaptic plasticity in PCs. Ryanodine receptors (RyRs) and IP_3_ receptors (IP_3_Rs) play key roles in Ca^2+^ release from the ER. This Ca^2+^ regulation is crucial for the function of cerebellar circuits, impacting motor learning, since the ER interacts with synaptic receptors, including AMPA and mGluRs (Konietzny et al., 2023). Synaptopodin is an actin-associated protein highly expressed in neuronal dendritic spines. It is known to organise the spine apparatus, a special form of the ER inside dendritic spines. It plays an important role in Ca^2+^ signalling and synaptic plasticity. Synaptopodin is mainly found in cortical PNs (especially in the hippocampus and neocortex), but it is not expressed in PCs. PCs have other types of ER structures in their spines (such as spine smooth ER) (Mundel et al., 1997; Deller et al., 2000; Vlachos et al., 2009; Wagner et al., 2011).

A recent investigation revealed that PIP_2_ can be primarily located in PC spines and GrC presynaptic active zones (Eguchi et al., 2023). As explained above, during glutamatergic stimulation, mGluR1 stimulates PLCβ to cleave PIP_2_ into DAG and IP_3_, which releases ER Ca^2+^ by activating IP_3_Rs. IP_3_-induced ER Ca^2+^ release can then be amplified by Ca^2+^-induced Ca^2+^ release (CICR) through RyRs and lead to a dramatic reduction in the ER Ca^2+^ concentration ([Ca^2+^]_ER_). Stromal interaction molecule (STIM) proteins, namely, STIM1 and STIM2, can, respectively, detect large and small decreases in the [Ca^2+^]_ER_; once activated, STIM proteins oligomerize and translocate to ER-plasma membrane junctions, known as *puncta*, where they bind to and gate the Ca^2+^-permeable channel, Orai1. This mechanism is known as store-operated Ca^2+^ entry (SOCE) and is primarily responsible for refilling ER Ca^2+^ in neurons (Moccia et al., 2015). STIM1 is abundantly expressed in cerebellar PCs (Klejman et al., 2009) and a recent investigation reported that it is preferentially localized in the dendritic subsurface cisterns of the ER in mouse PCs (Nomura et al., 2025). Orai1 is also highly expressed in cerebellar PCs from several species, including human, rat and Cynomolgus monkey (Guzman et al., 2014), but it is still unclear whether it contributes to SOCE. In this view, PCs are also enriched with Orai2 (Skibinska-Kijek et al., 2009), which may serve as a dominant negative regulator of Orai1 (Kito et al., 2015; Yoast et al., 2020), thereby strongly limiting Orai1-mediated Ca^2+^ entry in PCs. However, STIM1 can interact with many other components of the Ca^2+^ handling machinery (Moccia et al., 2015), including Cav1.2 channels (Wang et al., 2010), NMDA receptors (Gruszczynska-Biegala et al., 2020), AMPA receptors (Gruszczynska-Biegala et al., 2016), and members of Transient Receptor Potential (TRP) superfamily of non-selective cation channels, such as TRP Canonical 1 (TRPC1) and TRPC3 (Zeng et al., 2008; Lee et al., 2014). TRPC1 and TRPC3 are both expressed in PCs, but only TRPC3 can be gated by STIM1 in response to IP_3_-dependent ER Ca^2+^ release (Hartmann et al., 2008). A series of investigations has unambiguously demonstrated that STIM1-gated TRPC3-containing channels mediated mGluR1-dependent slow synaptic excitatory postsynaptic currents (EPSP_slow_) in cerebellar PCs (Hartmann et al., 2014; Gui et al., 2024). STIM1 maintains the ER Ca^2+^ pool that it mobilized during dendritic mGluR1 signalling to ensure motor coordination (Hartmann et al., 2014), regulates PC intrinsic excitability by interacting with Sarco-Endoplasmic Reticulum Ca^2+^-ATPase (SERCA) to clear intracellular Ca^2+^ and fine-tune the recruitment of Ca^2+^-dependent conductances (Ryu et al., 2017), and is crucial for the memory consolidation of the vestibulo-ocular reflex (Jang et al., 2020). The major expression of STIM1 and SERCA2 was detected at the dendritic level with little to no presence on spines. Conversely, two other critical receptors for the release of Ca^2+^ from the smooth ER (SER), RyR1 and IP_3_R1, were expressed on spines. The former had a lower spine expression compared to the somato-dendrites compartments, while the latter was highly expressed in spines (Nomura et al., 2025). An early study showed that the rapid replenishment of the ER Ca^2+^ store within the spine is driven by the intraluminal redistribution of dendritic Ca^2+^ (Okubo et al., 2015). This observation suggests that the ER within the spine neck does not represent a significant barrier to Ca^2+^ diffusion and that the absence of STIM1 impairs the overall ER Ca^2+^ dynamics in PCs. The neuronal ER functions act as an intracellular tunnel to redistribute stored Ca^2+^ within the neurons and as a leaky integrator of Ca^2+^ spike-inducing synaptic inputs (Okubo et al., 2015). This separation can lead to two distinct levels of synaptic plasticity; one strictly located on the dendritic level and one confined in each spine. This compartmentalized Ca^2+^ regulation is critical for cerebellar function and motor coordination.

#### Cytoskeleton

The cytoskeleton within the dendritic spines of PCs is primarily composed of filamentous actin (F-actin), which provides structural support and facilitates synaptic plasticity. Several key proteins regulate the organization and dynamics of this actin cytoskeleton: 1) Myosin XVI is a motor protein that interacts with the WAVE Regulatory Complex (WRC) to modulate actin dynamics in PC spines. Inhibition of the WRC accelerates F-actin turnover, resulting in altered spine morphology and reduced structural plasticity (Roesler et al., 2019). 2) Cortactin is predominantly localized near the postsynaptic density and sub-membrane regions of PC spines, and plays a role in actin filament branching and stabilization. Its distribution in these spines differs from that in forebrain neurons, suggesting region-specific functions in synaptic architecture (Szabó et al., 2021). 3) CaMKIIβ (Ca^2+^/Calmodulin-Dependent Protein Kinase II Beta) is the most abundant protein in the PSD and it is involved in synaptic plasticity through the phosphorylation of multiple NMDA subunits (Kennedy, 2000). This kinase also promotes spine formation and elongation through its F-actin binding activity (Okamoto et al., 2007). Activation of group I mGluRs, i.e., mGluR1 and mGluR5, triggers protein kinase C (PKC)-mediated phosphorylation of CaMKIIβ, leading to its dissociation from F-actin. This mechanism prevents excessive spine development and maintains proper spine morphology in mature PCs (Sugawara et al., 2017). 4) Myosin-Va is a motor protein responsible for transporting the ER into dendritic spines of PCs. The presence of ER in spines is essential for synaptic plasticity, and myosin-Va facilitates this process by pulling the ER into spines along actin filaments (Wagner et al., 2011).

These proteins collectively contribute to the dynamic regulation of the actin cytoskeleton in PC spines, influencing their structure and function in cerebellar synaptic plasticity (for a comparison, see Table 3).

**TABLE 3:** Comparison of ER related properties. Differences between cortex, hippocampus and Purkinje cells.

Feature	Pyramidal neuron (neocortex)	Pyramidal neuron (hippocampus)	Purkinje cell (cerebellum)
Spine Apparatus	Present (Space-filling ER structure) (Mundel et al., 1997; Deller et al., 2000)	Present complex multilamellar structure (Deller et al., 2000; Spacek and Harris, 1997)	Absent or extremely rare (Deller et al., 2000)
Smooth ER in Spines	Present, associated with spine apparatus (Deller et al., 2000)	Present, highly structured, contributes to spine apparatus (Spacek and Harris, 1997)	Present simpler tubular ER without a spine apparatus (Martone et al., 1993)
Synaptopodin Expression	High; essential for spine apparatus (Mundel et al., 1997; Deller et al., 2000)	High; essential for forming spine apparatus (Mundel et al., 1997; Deller et al., 2000)	Very low or absent (Deller et al., 2000)
Other Actin-Associated Proteins	Actin-binding proteins (e.g., α-actinin) (Mundel et al., 1997)	α-actinin, drebrin, important for actin/ER organization (Mundel et al., 1997)	Other cytoskeletal proteins; synaptopodin absent (Martone et al., 1996)
Main Function of ER	Ca^2+^ storage, buffering, and plasticity (supports LTP) (Spacek and Harris, 1997)	Ca^2+^ storage, modulation of synaptic plasticity (supports LTP) (Spacek and Harris, 1997)	Ca^2+^ buffering for LTD, especially after parallel fiber activation (Llano et al., 1991)
Synaptic Plasticity Linked to ER	Supports LTP (Spacek and Harris, 1997; Deller et al., 2000)	Supports LTP — local Ca^2+^ release needed for strengthening synapses (Spacek and Harris, 1997; Deller et al., 2000)	Supports LTD — local Ca^2+^ dynamics required for weakening synapses (Konnerth et al., 1992)
ER Complexity	Complex, multilayered spine apparatus (Spacek and Harris, 1997)	Highly complex, stacked cisternae (spine apparatus) (Spacek and Harris, 1997)	Simple, fine tubular ER network (Martone et al., 1993)
Calcium Release Mechanisms	IP3 receptors and ryanodine receptors on spine ER (Sharp et al., 1993)	IP3 receptors and ryanodine receptors on spine ER (Sharp et al., 1993)	IP3-mediated Ca^2+^ release; ryanodine receptors also present (Finch and Augustine, 1998)

## Purkinje cell spine regulation

### Parallel fibers (anti-Hebbian) and climbing fibers (Hebbian) long-term potentiation and depression

PC dendrites receive excitatory inputs from PFs and CFs and the spines are instrumental in generating specific forms of long-term synaptic plasticity, including long-term potentiation (LTP) and LTD. While synaptic plasticity at CF – spine synapses follows the Hebbian rules, the PF – spine synapse present both LTD and LTP based on a non-Hebbian plasticity rule (Roberts and Leen, 2010; Piochon et al., 2012; Runge et al., 2020). The general synaptic plasticity rule (Lisman, 1989; Shouval et al., 2002; Pali et al., 2025) dictates that LTP is generated by low Ca^2+^ concentrations and LTD by high concentrations. LTD at PF - PC synapses consists of an activity-dependent long-lasting reduction in synaptic strength (Roberts and Leen, 2010; Nishiyama and Yasuda, 2015). Coincidence of PF stimulation (glutamate release) and CF activation (membrane depolarisation and Ca^2+^ influx) triggers LTD (Piochon et al., 2012; Daida et al., 2024). This leads to an influx of Ca^2+^ via voltage-gated Ca^2+^ channels and to Ca^2+^ release from the endogenous ER stores through the CICR process (Harvey-Girard et al., 2010). Glutamate released from PFs activates mGluR1 to produce IP_3_ production, thereby promoting IP_3_-induced Ca^2+^ release from the spine apparatus (Hartmann et al., 2011). A high localised Ca^2+^ concentration, together with PKC activation, induces AMPA receptor (GluA2 subunit) internalisation from the postsynaptic membrane, weakening synaptic transmission (Lisman, 1989). LTD is essential for motor learning, such as eye-blink conditioning and adaptation of the vestibulo-ocular reflex (Sharp et al., 1993; Finch and Augustine, 1998; Hansel et al., 2001; Ito, 2001). Moderate PF activation without strong CF co-activation leads to protein kinase A (PKA) stimulation and enhances AMPA receptor phosphorylation, promoting their insertion or stabilisation at the postsynaptic membrane, involving the activation of phosphatases, such as protein phosphatase 1 (PP1) and PP2B (calcineurin) (Lewis and Maler, 2002). The nitric oxide (NO)/soluble guanylyl cyclase/cyclinc guanosine monophosphate (cGMP) signalling pathway has also been implicated (Lev-Ram et al., 2002). Unlike LTD, where a large, spatially localised Ca^2+^ rise triggers depression, LTP requires smaller, slower Ca^2+^ elevations that fails to engage the higher threshold LTD pathway. LTP may help counterbalance LTD, maintaining synaptic homeostasis and contributing to fine-tuning of motor commands (Martone et al., 1993; Spacek and Harris, 1997; Lev-Ram et al., 2002; Coesmans et al., 2004). The presynaptic protein RIM1, in connection with Rab3-interacting molecule, is necessary for LTP to occur between PFs and PC (Uriu et al., 2010). It should also be noted that also presynaptic forms of synaptic plasticity also exist at the PF-PC synapse but are not considered here (Hansel et al., 2001).

#### How to modulate a spine: presynaptic release probability

There are various substances that can module the overall synaptic strength without the need to physically eliminate the PF-spine synapse. The endocannabinoids, which are produced by PCs and act as a retrograde signal, interact with the presynaptic side, reducing the release probability through CB1 receptors (Safo et al., 2006). A critical presynaptic protein termed RIM1 is important in the control and recruitment of presynaptic Ca^2+^ channels (Kaeser and Regehr, 2014). This protein is activated by progranulin generated by PCs and, acting as a diffusible signal, leads instead to an increase in release probability. This strengthening of the synaptic activity was recorded during the stage in which a CF becomes the winner with its translocation from somatic to dendritic spines (Uriu et al., 2010; Nitta et al., 2025). The presynaptic NMDA receptors are involved in the production of NO, which, compared to many other neuronal types, is not produced by the postsynaptic side (D’Angelo, 2014; Mapelli et al., 2017). NO may influence the postsynaptic Ca^2+^ dynamics and thereby change the overall strength of the presynaptic side, pushing the synapse into LTP (Schonewille et al., 2021). The structural proteins between PF and spines are critical, but the axon itself can define if a presynaptic active site needs to be stabilized or abolished (Aiken and Holzbaur, 2024). Based on the type of signals that need to be elaborated by a specific PCs, it is possible that the synapse is initially established between PF and PC and a certain point in the development, the presynaptic side itself is pruned.

#### How to modulate a spine: postsynaptic properties

As previously defined (see chapter “Spine number estimates”), to reduce the impact of the large number of spines and their low response it was assumed the absence of fast AMPA receptor-mediated responses, and the presence of a majority of mGluR1/TRPC3-mediated slow synaptic currents (Jin et al., 2007). These slow EPSC, lasting up to hundreds of milliseconds, were recently shown to change greatly depending on the lobuli (Thomas et al., 2024). Slow responses could be elicited in spines with only one head to preserve the PF-spine synapse, to convey support information, or to maintain the synapse active when no relevant information is transmitted. This model is supported by the recent discovery that branched spines are more “eloquent” compared to single-headed spines (Loschky et al., 2022). Another way to reduce the number spines, without physical deletion, is through GABAB receptors located peri synaptically and on spine necks. These receptors are connected with GIRK2 channels that can act as a filter for small intensity presynaptic activation or by slow responses elicited by mGluR1. The PF – spine synapse follows an anti-Hebbian rule to generate short and long-term potentiation (Lev-Ram et al., 2002; Piochon et al., 2012). This is in agreement with the evidence that postsynaptic Ca^2+^ needs to remain low to generate LTP, while it must increase by coincident activation of CFs to generate LTD.

### Dynamic changes in morphological conformation

A property that was studied in layer 5 PN showed that the length of spine necks electrically isolates the heads from the dendrites. This activity was recorded using Spine Uncaging Potentials and showed a correlation between the neck lengths and electrical activity recorded at the somatic level. Longer necks had more impact on the activity, even reaching a complete silencing of the post-synaptic potentials, where shorted neck allowed post-synaptic potential transmission (Araya et al., 2006). When a synaptic contact is established, its shape does not change even during LTD activity (Sdrulla and Linden, 2007). This view was recently challenged with a new experimental procedure showing that, besides changing their shape, the entire dendritic spine can be retracted and regenerated along the day/night cycle (Loschky et al., 2022). An *in vivo* experimental procedure further showed that PC spine size can be changed by a process that required the endocytosis of some spine membrane by BG (Morizawa et al., 2022). This process was marked by increased activity of BGs after training and learning.

### Spine modification in diseases

Spine properties can be greatly modified in the presence of mutations that dysregulate various signalling pathways, thereby resulting in severe neurological diseases. In many cases, the PC dendritic tree can change its shape, branching points and overall arborisation. The dendritic trees are subject to marked modifications as exemplified by the atrophy observed in PCs of Weaver mice and in ectopic PCs of Reeler mice. In both mouse models, the presence of spines is unaffected by the mutations, but their linear count is lower compared to controls (Yuste and Bonhoeffer, 2004). In essential tremor, a human parkinsonism characterized by localised axon swelling, there is a reduction in the complexity of PC dendritic tree with just a small reduction in spine number per unit length (Louis et al., 2014). In Staggerer mice, which are missing the retinoid-related orphan receptor α (RORα), the animal is ataxic (SCA1) and PCs show stunted trees with parts of them completely devoid of spines (Mitsumura et al., 2011). Some remnants of the PF-PC connectivity can be observed with excitatory synapses made directly on the dendritic surface. Another ataxia (SCA2) is caused by polyglutamine expansion in Ataxin-2 (ATXN2) and its activity on CaMKIIα and CaMKIV signalling with a reduction in spine length and spine density (Arsović et al., 2020). A point mutation in the protein kinase C gamma (PKCγ), involved in SCA14, showed that it has a limited impact on spinogenesis except if it is upregulated. In the latter case, it causes the reduction of the number of spines, their length and overall maturity (Sziber et al., 2025). In human schizophrenia, a decrease in spine density was observed, but with no information about changes to the spine shape (Mavroudis et al., 2017). This specific mutation is yet to be replicated in animal models.

## Conclusions and computational implications

Although the number of spines appears to be lower than initially thought, this is not expected to hamper the encoding capabilities of PCs. Indeed, instead of having ∼90% silent synapses out of 100000, there would be a maximum of 85% inactive or poorly active synapse out of 35,000. The more “eloquent” double-headed spines would be 15% of the total, i.e., ∼5,000 in mouse and ∼40,000 in human PCs contribute more to the modularity of cerebellar organisation (Streng et al., 2025) compared to the single-headed spines. We recently compared PCs in mice and humans (Masoli et al., 2024), showing that the human/mouse spine head ratio (7.5) could determine the computing capability of the neurons. This number compared well with other metrics like the dendritic surface ratio (5.5) and dendritic complexity index (6.5), as well as with dendritic transfer impedance computed for clusters of spines that can effectively impact spike generation in the PC axonal initial segment (6.5) with 1-ms time resolution. This suggested that the increased number of contacts was almost entirely transformed into effective combinations of input patterns that can regulate spike generation in the soma, akin to the linear encoding in a perceptron (Brunel et al., 2004; Walter et al., 2009). The maximum computational capacity, which depends on the number of alternative states established by the dendrites, turned out to be 2^8^ for mice and 2^51^ for human (Masoli et al., 2024). It remains to be determined whether these figures would change by making assumptions about spine efficiency, e.g., following the arguments reported here. The electrical isolation generated by the spine neck, the large number of thin spines, and the hyperpolarizing activity of GABAB/GIRK2 channels suggest that each spine may individually influence the overall neuronal encoding activity. This is because of the reduction in the noise/signal ratio, allowing the transmission of strong excitatory activity concentrated on few spines. It should be noted that, owing to the redundancy of dendritic combinations, some output spike patterns may be mutually indistinguishable on the temporal resolution scale of the neuron. Ad hoc simulations using PC computational models with spines may allow the calculation of the combinatorial capacity in human and mouse PCs under more realistic assumptions, for example, that segments are not fully active or inactive or that spine independence is incomplete, or that individual spines have specific and differentiated neurotransmission properties reflecting modulatory, plastic, or pathological states (Rieke, 1999; London et al., 2002; Arleo et al., 2010).

## References

[B1] Adams P. Rungta R. Garcia E. van den Maagdenberg A. M. J. M. MacVicar B. A. Snutch T. P. (2010). Contribution of calcium-dependent facilitation to synaptic plasticity revealed by migraine mutations in the P/Q-type calcium channel. Proc. 107, 18694–18699. 10.1073/pnas.1009500107 20937883 PMC2972937

[B2] Aiken J. Holzbaur E. L. F. (2024). Spastin locally amplifies microtubule dynamics to pattern the axon for presynaptic cargo delivery. Curr. Biol. 34, 1687–1704.e8. 10.1016/j.cub.2024.03.010 38554708 PMC11042977

[B3] Aimi T. Matsuda K. Yuzaki M. (2023). C1ql1-Bai3 signaling is necessary for climbing fiber synapse formation in mature Purkinje cells in coordination with neuronal activity. Mol. Brain 16, 61–17. 10.1186/s13041-023-01048-4 37488606 PMC10367388

[B4] Al-Hussain S. M. Yousuf M. S. Hani A. B. Zaqout S. Djouhri L. Mustafa A. G. (2022). A Golgi study of neurons in the camel cerebellum (*Camelus dromedarius*). Anat. Rec. 305, 1264–1276. 10.1002/ar.24742 34390196

[B5] Albus J. S. (1971). A theory of cerebellar function. Math. Biosci. 10, 25–61. 10.1016/0025-5564(71)90051-4

[B6] Alfaro-Ruíz R. Aguado C. Martín-Belmonte A. Moreno-Martínez A. E. Luján R. (2020). Cellular and subcellular localisation of kv4-associated kchip proteins in the rat cerebellum. Int. J. Mol. Sci. 21, 6403–6419. 10.3390/ijms21176403 32899153 PMC7503578

[B7] Araya R. Jiang J. Eisenthal K. B. Yuste R. (2006). The spine neck filters membrane potentials. Proc. Natl. Acad. Sci. U. S. A. 103, 17961–17966. 10.1073/pnas.0608755103 17093040 PMC1693855

[B8] Arleo A. Nieus T. Bezzi M. D’Errico A. D’Angelo E. Coenen O. (2010). How synaptic release probability shapes neuronal transmission: information-theoretic analysis in a cerebellar granule cell. Neural comput. 22, 2031–2058. 10.1162/NECO_a_00006-Arleo 20438336

[B9] Arsović A. Halbach M. V. Canet-Pons J. Esen-Sehir D. Döring C. Freudenberg F. (2020). Mouse ataxin-2 expansion downregulates camkii and other calcium signaling factors, impairing granule—purkinje neuron synaptic strength. Int. J. Mol. Sci. 21, 6673–36. 10.3390/ijms21186673 32932600 PMC7555182

[B10] Bao D. Pang Z. Morgan M. A. Parris J. Rong Y. Li L. (2006). Cbln1 is essential for interaction-dependent secretion of Cbln3. Mol. Cell. Biol. 26, 9327–9337. 10.1128/mcb.01161-06 17030622 PMC1698530

[B11] Bentivoglio M. Cotrufo T. Ferrari S. Tesoriero C. Mariotto S. Bertini G. (2019). The original histological slides of camillo golgi and his discoveries on neuronal structure. Front. Neuroanat. 13, 3–13. 10.3389/fnana.2019.00003 30833889 PMC6388087

[B12] Benton M. D. Raman I. M. (2009). Stabilization of Ca current in Purkinje neurons during high-frequency firing by a balance of Ca-dependent facilitation and inactivation. Channels (Austin). 3, 393–401. 10.4161/chan.3.6.9838 19806011 PMC2897944

[B13] Boele H. J. Peter S. Ten Brinke M. M. Verdonschot L. Ijpelaar A. C. H. Rizopoulos D. (2018). Impact of parallel fiber to Purkinje cell long-term depression is unmasked in absence of inhibitory input. Sci. Adv. 4, eaas9426–eaas9429. 10.1126/sciadv.aas9426 30306129 PMC6170036

[B14] Boivin J. R. Nedivi E. (2018). Functional implications of inhibitory synapse placement on signal processing in pyramidal neuron dendrites. Curr. Opin. Neurobiol. 51, 16–22. 10.1016/j.conb.2018.01.013 29454834 PMC6066407

[B15] Brunel N. Hakim V. Isope P. Nadal J. P. Barbour B. (2004). Optimal information storage and the distribution of synaptic weights: perceptron versus Purkinje cell. Neuron 43, 745–757. 10.1016/j.neuron.2004.08.023 15339654

[B16] Brunetti V. Soda T. Berra-Romani R. De Sarro G. Guerra G. Scarpellino G. (2024). Two signaling modes are better than one: flux-independent signaling by ionotropic glutamate receptors is coming of age. Biomedicines 12, 880. 10.3390/biomedicines12040880 38672234 PMC11048239

[B17] Buceta I. Elezgarai I. Rico-Barrio I. Gerrikagoitia I. Puente N. Grandes P. (2020). Deletion of the cannabinoid CB1 receptor impacts on the ultrastructure of the cerebellar parallel fiber-Purkinje cell synapses. J. Comp. Neurol. 528, 1041–1052. 10.1002/cne.24808 31721187

[B18] Burada A. P. Vinnakota R. Kumar J. (2020). The architecture of GluD2 ionotropic delta glutamate receptor elucidated by cryo-EM. J. Struct. Biol. 211, 107546. 10.1016/j.jsb.2020.107546 32512155

[B19] Burada A. P. Vinnakota R. Bharti P. Dutta P. Dubey N. Kumar J. (2022). Emerging insights into the structure and function of ionotropic glutamate delta receptors. Br. J. Pharmacol. 179, 3612–3627. 10.1111/bph.15313 33145757

[B20] Busch S. E. Hansel C. (2023). Climbing fiber multi-innervation of mouse Purkinje dendrites with arborization common to human. Science 381, 420–427. 10.1126/science.adi1024 37499000 PMC10962609

[B21] Busch S. E. Hansel C. (2025). eLife Assessment: non-allometric expansion and enhanced compartmentalization of Purkinje cell dendrites in the human cerebellum. 10.7554/eLife.105013.2.sa3 PMC11999696 40231436

[B22] Campeau J. L. Wu G. Bell J. R. Rasmussen J. Sim V. L. (2013). Early increase and late decrease of Purkinje cell dendritic spine density in prion-infected organotypic mouse cerebellar cultures. PLoS One 8, e81776–e81777. 10.1371/journal.pone.0081776 24312586 PMC3847088

[B23] Castejón O. J. Dailey M. E. (2009). Immunohistochemistry of GluR1 subunits of AMPA receptors of rat cerebellar nerve cells. Biocell 33, 71–80. 10.32604/biocell.2009.33.071 19886034

[B24] Chen X. Du Y. Broussard G. J. Kislin M. Yuede C. M. Zhang S. (2022). Transcriptomic mapping uncovers Purkinje neuron plasticity driving learning. Nature 605, 722–727. 10.1038/s41586-022-04711-3 35545673 PMC9887520

[B25] Coesmans M. Weber J. T. De Zeeuw C. I. Hansel C. (2004). Bidirectional parallel fiber plasticity in the cerebellum under climbing fiber control. Neuron 44, 691–700. 10.1016/j.neuron.2004.10.031 15541316

[B26] Cramer S. Gao W. Chen G. Ebner T. J. (2013). Reevaluation of the beam and radial hypotheses of parallel fiber action in the cerebellar cortex. J. Neurosci. 33, 11412–11424. 10.1523/JNEUROSCI.0711-13.2013 23843513 PMC3724546

[B27] Crépe F. Galante M. Habbas S. McLean H. Danie H. (2011). Role of the vesicular transporter VGLUT3 in retrograde release of glutamate by cerebellar Purkinje cells. J. Neurophysiol. 105, 1023–1032. 10.1152/jn.00736.2010 21177991

[B28] Daida A. Kurotani T. Yamaguchi K. Takahashi Y. Ichinohe N. (2024). Different numbers of conjunctive stimuli induce LTP or LTD in mouse cerebellar purkinje cell. Cerebellum 23, 2297–2307. 10.1007/s12311-024-01726-6 39096432 PMC11585524

[B29] Davidson A. M. Mejiá-Gómez H. Jacobowitz M. Mostany R. (2020). Dendritic spine density and dynamics of layer 5 pyramidal neurons of the primary motor cortex are elevated with aging. Cereb. Cortex 30, 767–777. 10.1093/cercor/bhz124 31298696 PMC7306167

[B30] Defelipe J. (2025). Cajal and the discovery of the Golgi method: a neuroanatomist ’ s dream. Anat. Sci. Int. 10.1007/s12565-025-00840-7 40323529 PMC12513909

[B31] Dehnes Y. Chaudhry F. A. Ullensvang K. Lehre K. P. Storm-Mathisen J. Danbolt N. C. (1998). The glutamate transporter EAAT4 in rat cerebellar Purkinje cells: a glutamate-gated chloride channel concentrated near the synapse in parts of the dendritic membrane facing astroglia. J. Neurosci. 18, 3606–3619. 10.1523/jneurosci.18-10-03606.1998 9570792 PMC6793133

[B32] Deller T. Merten T. Roth S. U. Mundel P. Frotscher M. (2000). Actin-associated protein synaptopodin in the rat hippocampal formation: localization in the spine neck and close association with the spine apparatus of principal neurons. J. Comp. Neurol. 418, 164–181. 10.1002/(SICI)1096-9861(20000306)418:2<164::AID-CNE4>3.0.CO;2-0 10701442

[B33] Desmond J. E. Fiez J. A. (1998). Neuroimaging studies of the cerebellum: language, learning and memory. Trends Cogn. Sci. 2, 355–362. 10.1016/S1364-6613(98)01211-X 21227232

[B34] Dusart I. Flamant F. (2012). Profound morphological and functional changes of rodent Purkinje cells between the first and the second postnatal weeks: a metamorphosis? Front. Neuroanat. 6, 11. 10.3389/fnana.2012.00011 22514522 PMC3324107

[B35] D’Angelo E. (2014). The organization of plasticity in the cerebellar cortex: from synapses to control. 1st ed. Elsevier B.V. 10.1016/B978-0-444-63356-9.00002-9 24916288

[B36] Egorova P. A. Marinina K. S. Bezprozvanny I. B. (2023). Chronic suppression of STIM1-mediated calcium signaling in Purkinje cells rescues the cerebellar pathology in spinocerebellar ataxia type 2. Biochim. Biophys. Acta - Mol. Cell Res. 1870, 119466. 10.1016/j.bbamcr.2023.119466 36940741

[B37] Eguchi K. Le Monnier E. Shigemoto R. (2023). Nanoscale phosphoinositide distribution on cell membranes of mouse cerebellar neurons. J. Neurosci. 43, 4197–4216. 10.1523/JNEUROSCI.1514-22.2023 37160366 PMC10255094

[B38] Finch E. A. Augustine G. J. (1998). Local calcium signalling by inositol-1,4,5-trisphosphate in Purkinje cell dendrites. Nature 396, 753–756. 10.1038/25541 9874372

[B39] Fuccillo M. V. Földy C. Gökce Ö. Rothwell P. E. Sun G. L. Malenka R. C. (2015). Single-cell mRNA profiling reveals cell-type-specific expression of neurexin isoforms. Neuron 87, 326–340. 10.1016/j.neuron.2015.06.028 26182417 PMC4733560

[B40] Furutani K. Okubo Y. Kakizawa S. Iino M. (2006). Postsynaptic inositol 1,4,5-trisphosphate signaling maintains presynaptic function of parallel fiber-Purkinje cell synapses via BDNF. Proc. Natl. Acad. Sci. U. S. A. 103, 8528–8533. 10.1073/pnas.0600497103 16709674 PMC1482525

[B41] Gao Y. Perkins E. M. Clarkson Y. L. Tobia S. Lyndon A. R. Jackson M. (2011). β-III spectrin is critical for development of purkinje cell dendritic tree and spine morphogenesis. J. Neurosci. 31, 16581–16590. 10.1523/JNEUROSCI.3332-11.2011 22090485 PMC3374928

[B42] Gelfo F. Florenzano F. Foti F. Burello L. Petrosini L. De Bartolo P. (2016). Lesion-induced and activity-dependent structural plasticity of Purkinje cell dendritic spines in cerebellar vermis and hemisphere. Brain Struct. Funct. 221, 3405–3426. 10.1007/s00429-015-1109-5 26420278

[B43] Gilbert M. Rasmussen A. (2025). The cerebellar deep nuclei: a patch for rate codes? Front. Neural Circuits 19, 1548123–14. 10.3389/fncir.2025.1548123 40265048 PMC12011825

[B44] Golgi C. (1883). Sulla fina anatomia degli organi centrali del sistema nervoso. Riv. Sper. Freniatr.

[B45] Gruszczynska-Biegala J. Sladowska M. Kuznicki J. (2016). AMPA receptors are involved in store-operated calcium entry and interact with STIM proteins in rat primary cortical neurons. Front. Cell. Neurosci. 10, 251. 10.3389/fncel.2016.00251 27826230 PMC5078690

[B46] Gruszczynska-Biegala J. Strucinska K. Maciag F. Majewski L. Sladowska M. Kuznicki J. (2020). STIM protein-NMDA2 receptor interaction decreases NMDA-dependent calcium levels in cortical neurons. Cells 9, 160. 10.3390/cells9010160 31936514 PMC7017226

[B47] Gui L. Tellios V. Xiang Y.-Y. Feng Q. Inoue W. Lu W.-Y. (2024). Neuronal nitric oxide synthase regulates cerebellar parallel fiber slow EPSC in purkinje neurons by modulating STIM1-gated TRPC3-containing channels. Cerebellum 23, 1867–1881. 10.1007/s12311-024-01683-0 38472628

[B48] Guzman R. Valente E. G. Pretorius J. Pacheco E. Qi M. Bennett B. D. (2014). Expression of ORAII, a plasma membrane resident subunit of the CRAC channel, in rodent and non-rodent species. J. Histochem. Cytochem. 62, 864–878. 10.1369/0022155414554926 25249026 PMC4244304

[B49] Han P. She Y. Yang Z. Zhuang M. Wang Q. Luo X. (2022). Cbln1 regulates axon growth and guidance in multiple neural regions. PLOS Biol. 20, e3001853. 10.1371/journal.pbio.3001853 36395107 PMC9671368

[B50] Hansel C. Linden D. J. D’Angelo E. (2001). Beyond parallel fiber LTD: the diversity of synaptic and non-synaptic plasticity in the cerebellum. Nat. Neurosci. 4, 467–475. 10.1038/87419 11319554

[B51] Hansen K. B. Naur P. Kurtkaya N. L. Kristensen A. S. Gajhede M. Kastrup J. S. (2009). Modulation of the dimer interface at ionotropic glutamate-like receptor delta2 by D-serine and extracellular calcium. J. Neurosci. 29, 907–917. 10.1523/JNEUROSCI.4081-08.2009 19176800 PMC2806602

[B52] Harris K. M. Stevens J. K. (1988). Dendritic spines of rat cerebellar Purkinje cells: serial electron microscopy with reference to their biophysical characteristics. J. Neurosci. 8, 4455–4469. 10.1523/JNEUROSCI.08-12-04455.1988 3199186 PMC6569567

[B53] Hartmann J. Dragicevic E. Adelsberger H. Henning H. A. Sumser M. Abramowitz J. (2008). TRPC3 channels are required for synaptic transmission and motor coordination. Neuron 59, 392–398. 10.1016/j.neuron.2008.06.009 18701065 PMC2643468

[B54] Hartmann J. Henning H. A. Konnerth A. (2011). mGluR1/TRPC3-mediated synaptic transmission and calcium signaling in mammalian central neurons. Cold Spring Harb. Perspect. Biol. 3, a006726–16. 10.1101/cshperspect.a006726 21441586 PMC3062210

[B55] Hartmann J. Karl R. M. Alexander R. P. D. Adelsberger H. Brill M. S. Rühlmann C. (2014). STIM1 controls neuronal Ca^2+^ signaling, mGluR1-dependent synaptic transmission, and cerebellar motor behavior. Neuron 82, 635–644. 10.1016/j.neuron.2014.03.027 24811382

[B56] Harvey-Girard E. Lewis J. Maler L. (2010). Burst-induced anti-hebbian depression acts through short-term synaptic dynamics to cancel redundant sensory signals. J. Neurosci. 30, 6152–6169. 10.1523/JNEUROSCI.0303-10.2010 20427673 PMC6632600

[B57] Heck D. Sultan F. (2002). Cerebellar structure and function: making sense of parallel fibers. Hum. Mov. Sci. 21, 411–421. 10.1016/s0167-9457(02)00123-9 12381396

[B58] Heintz T. G. Eva R. Fawcett J. W. (2016). Regional regulation of purkinje cell dendritic spines by integrins and Eph/ephrins. PLoS One 11, e0158558–15. 10.1371/journal.pone.0158558 27518800 PMC4982633

[B59] Hibi M. Matsuda K. Takeuchi M. Shimizu T. Murakami Y. (2017). Evolutionary mechanisms that generate morphology and neural-circuit diversity of the cerebellum. Dev. Growth Differ. 59, 228–243. 10.1111/dgd.12349 28470724

[B60] Hills L. B. Masri A. Konno K. Kakegawa W. Lam A. T. N. Lim-Melia E. (2013). Deletions in GRID2 lead to a recessive syndrome of cerebellar ataxia and tonic upgaze in humans. Neurology 81, 1378–1386. 10.1212/WNL.0b013e3182a841a3 24078737 PMC3806907

[B61] Houston C. M. Diamanti E. Diamantaki M. Kutsarova E. Cook A. Sultan F. (2017). Exploring the significance of morphological diversity for cerebellar granule cell excitability. Sci. Rep. 7, 46147. 10.1038/srep46147 28406156 PMC5390267

[B62] Hoxha E. Tempia F. Lippiello P. Miniaci M. C. (2016). Modulation, plasticity and pathophysiology of the parallel fiber-purkinje cell synapse. Front. Synaptic Neurosci. 8, 35–16. 10.3389/fnsyn.2016.00035 27857688 PMC5093118

[B63] Hoxha E. Gabriele R. M. C. Balbo I. Ravera F. Masante L. Zambelli V. (2017). Motor deficits and cerebellar atrophy in Elovl5 knock out mice. Front. Cell. Neurosci. 11, 343–11. 10.3389/fncel.2017.00343 29163054 PMC5670146

[B64] Hoxha E. Balbo I. Miniaci M. C. Tempia F. (2018). Purkinje cell signaling deficits in animal models of ataxia. Front. Synaptic Neurosci. 10, 6–17. 10.3389/fnsyn.2018.00006 29760657 PMC5937225

[B65] Huang C. ming Huang R. H. (1998). Measuring parallel fiber length in the rat cerebellum. Brain Res. 801, 211–215. 10.1016/S0006-8993(98)00444-2 9729391

[B252] Huang C. Gammon S. J. Dieterle M. Huang R. H. Likins L. Ricklefs R. E. (2014). Dramatic increases in number of cerebellar granule-cell-Purkinje-cell synapses across several mammals. Mamm. Biol. 79, 163–169. 10.1016/j.mambio.2013.12.003

[B66] Huang M. Verbeek D. S. (2019). Why do so many genetic insults lead to Purkinje Cell degeneration and spinocerebellar ataxia? Neurosci. Lett. 688, 49–57. 10.1016/j.neulet.2018.02.004 29421540

[B67] Huang T.-Y. Lin L.-S. Cho K.-C. Chen S.-J. Kuo Y. Yu L. (2012). Chronic treadmill exercise in rats delicately alters the Purkinje cell structure to improve motor performance and toxin resistance in the cerebellum. J. Appl. Physiol. 113, 889–895. 10.1152/japplphysiol.01363.2011 22837167

[B68] Ichikawa R. Yamasaki M. Miyazaki T. Konno K. Hashimoto K. Tatsumi H. (2011). Developmental switching of perisomatic innervation from climbing fibers to basket cell fibers in cerebellar Purkinje cells. J. Neurosci. 31, 16916–16927. 10.1523/JNEUROSCI.2396-11.2011 22114262 PMC6623856

[B69] Ichikawa R. Sakimura K. Watanabe M. (2016). GluD2 endows parallel fiber-purkinje cell synapses with a high regenerative capacity. J. Neurosci. 36, 4846–4858. 10.1523/JNEUROSCI.0161-16.2016 27122040 PMC6601726

[B70] Iijima T. Miura E. Matsuda K. Kamekawa Y. Watanabe M. Yuzaki M. (2007). Characterization of a transneuronal cytokine family Cbln - regulation of secretion by heteromeric assembly. Eur. J. Neurosci. 25, 1049–1057. 10.1111/j.1460-9568.2007.05361.x 17331201

[B71] Indriati D. W. Kamasawa N. Matsui K. Meredith A. L. Watanabe M. Shigemoto R. (2013). Quantitative localization of Cav2.1 (P/Q-type) voltage-dependent calcium channels in Purkinje cells: somatodendritic gradient and distinct somatic coclustering with calcium-activated potassium channels. J. Neurosci. 33, 3668–3678. 10.1523/JNEUROSCI.2921-12.2013 23426693 PMC4031662

[B72] Isaac J. T. R. R. Ashby M. McBain C. J. (2007). The role of the GluR2 subunit in AMPA receptor function and synaptic plasticity. Neuron 54, 859–871. 10.1016/j.neuron.2007.06.001 17582328

[B73] Isope P. Barbour B. (2002). Properties of unitary granule cell Purkinje cell synapses in adult rat cerebellar slices. J. Neurosci. 22, 9668–9678. 10.1523/JNEUROSCI.22-22-09668.2002 12427822 PMC6757845

[B74] Ito M. (2001). Cerebellar long-term depression: characterization, signal transduction, and functional roles. Physiol. Rev. 81, 1143–1195. 10.1152/physrev.2001.81.3.1143 11427694

[B75] Ito-Ishida A. Kakegawa W. Kohda K. Miura E. Okabe S. Yuzaki M. (2014a). Cbln1 downregulates the formation and function of inhibitory synapses in mouse cerebellar Purkinje cells. Eur. J. Neurosci. 39, 1268–1280. 10.1111/ejn.12487 24467251

[B76] Ito-Ishida A. Okabe S. Yuzaki M. (2014b). The role of Cbln1 on Purkinje cell synapse formation. Neurosci. Res. 83, 64–68. 10.1016/j.neures.2014.01.009 24607546

[B77] Itoh M. Yuzaki M. (2024). The hidden face of GluD1 at inhibitory synapses. Cell Res. 34, 405–406. 10.1038/s41422-024-00931-6 38263278 PMC11143318

[B78] Jacobs B. Johnson N. L. Wahl D. Schall M. Maseko B. C. Lewandowski A. (2014). Comparative neuronal morphology of the cerebellar cortex in afrotherians, carnivores, cetartiodactyls, and primates. Front. Neuroanat. 8, 24. 10.3389/fnana.2014.00024 24795574 PMC4005950

[B79] Jahncke J. N. Schnell E. Wright K. M. (2025). Distinct functional domains of Dystroglycan regulate inhibitory synapse formation and maintenance in cerebellar Purkinje cells. Commun. Biol. 8, 878. 10.1038/s42003-025-08323-1 40473926 PMC12141699

[B80] Jang M. Bum Um K. Jang J. Jin Kim H. Cho H. Chung S. (2015). Coexistence of glutamatergic spine synapses and shaft synapses in substantia nigra dopamine neurons. Sci. Rep. 5, 14773–16. 10.1038/srep14773 26435058 PMC4593176

[B81] Jang D. C. Shim H. G. Kim S. J. (2020). Intrinsic plasticity of cerebellar purkinje cells contributes to motor memory consolidation. J. Neurosci. 40, 4145–4157. 10.1523/JNEUROSCI.1651-19.2020 32295816 PMC7244189

[B82] Jin Y. Kim S. J. Kim J. Worley P. F. Linden D. J. (2007). Long-term depression of mGluR1 signaling. Neuron 55, 277–287. 10.1016/j.neuron.2007.06.035 17640528 PMC2063510

[B83] Joensuu M. Wallis T. P. Saber S. H. Meunier F. A. (2020). Phospholipases in neuronal function: a role in learning and memory? J. Neurochem. 153, 300–333. 10.1111/jnc.14918 31745996

[B84] Jörntell H. Hansel C. (2006). Synaptic memories upside down: bidirectional plasticity at cerebellar parallel fiber-purkinje cell synapses. Neuron 52, 227–238. 10.1016/j.neuron.2006.09.032 17046686

[B85] Kaeser P. S. Regehr W. G. (2014). Molecular mechanisms for synchronous, asynchronous, and spontaneous neurotransmitter release. Annu. Rev. Physiol. 76, 333–363. 10.1146/annurev-physiol-021113-170338 24274737 PMC4503208

[B86] Kakegawa W. Miyazaki T. Kohda K. Matsuda K. Emi K. Motohashi J. (2009). The N-terminal domain of GluD2 (GluRdelta2) recruits presynaptic terminals and regulates synaptogenesis in the cerebellum *in vivo* . J. Neurosci. 29, 5738–5748. 10.1523/JNEUROSCI.6013-08.2009 19420242 PMC6665243

[B87] Kakegawa W. Miyoshi Y. Hamase K. Matsuda S. Matsuda K. Kohda K. (2011). D-Serine regulates cerebellar LTD and motor coordination through the δ glutamate receptor. Nat. Neurosci. 14, 603–613. 10.1038/nn.2791 21460832

[B88] Kennedy M. B. (2000). Signal-processing machines at the postsynaptic density. Sci. 80 290, 750–754. 10.1126/science.290.5492.750 11052931

[B89] Khan M. Z. (2017). Ionotropic glutamate receptors (iGluRs) of the delta family (GluD1 and GluD2) and synaptogenesis. Alex. J. Med. 53, 201–206. 10.1016/j.ajme.2016.09.003

[B90] Khouri-Farah N. Guo Q. Perry T. A. Dussault R. Li J. Y. H. (2025). FOXP genes regulate Purkinje cell diversity and cerebellar morphogenesis. Nat. Neurosci. 4, 1–21. 10.1038/s41593-025-02042-w 40826298 PMC12497650

[B91] Kim E. Y. Rumpf C. H. Fujiwara Y. Cooley E. S. Van Petegem F. Minor D. L. (2008). Structures of CaV2 Ca2+/CaM-IQ domain complexes reveal binding modes that underlie calcium-dependent inactivation and facilitation. Structure 16, 1455–1467. 10.1016/j.str.2008.07.010 18940602 PMC2701236

[B93] Kito H. Yamamura H. Suzuki Y. Yamamura H. Ohya S. Asai K. (2015). Regulation of store-operated Ca2+ entry activity by cell cycle dependent up-regulation of Orai2 in brain capillary endothelial cells. Biochem. Biophys. Res. Commun. 459, 457–462. 10.1016/j.bbrc.2015.02.127 25748572

[B94] Klejman M. E. Gruszczynska-Biegala J. Skibinska-Kijek A. Wisniewska M. B. Misztal K. Blazejczyk M. (2009). Expression of STIM1 in brain and puncta-like co-localization of STIM1 and ORAI1 upon depletion of Ca(2+) store in neurons. Neurochem. Int. 54, 49–55. 10.1016/j.neuint.2008.10.005 19013491

[B95] Konietzny A. Wegmann S. Mikhaylova M. (2023). The endoplasmic reticulum puts a new spin on synaptic tagging. Trends Neurosci. 46, 32–44. 10.1016/j.tins.2022.10.012 36428191

[B96] Konnerth A. Dreessen J. Augustine G. J. (1992). Brief dendritic calcium signals initiate long-lasting synaptic depression in cerebellar Purkinje cells. Proc. Natl. Acad. Sci. U. S. A. 89, 7051–7055. 10.1073/pnas.89.15.7051 1323125 PMC49643

[B97] Konno K. Matsuda K. Nakamoto C. Uchigashima M. Miyazaki T. Yamasaki M. (2014). Enriched expression of GluD1 in higher brain regions and its involvement in parallel fiber-interneuron synapse formation in the cerebellum. J. Neurosci. 34, 7412–7424. 10.1523/JNEUROSCI.0628-14.2014 24872547 PMC6795243

[B98] Kumar A. Paeger L. Kosmas K. Kloppenburg P. Noegel A. A. Peche V. S. (2016). Neuronal actin dynamics, spine density and neuronal dendritic complexity are regulated by CAP2. Front. Cell. Neurosci. 10, 180–17. 10.3389/fncel.2016.00180 27507934 PMC4960234

[B99] Kuo S.-H. Faust P. L. Vonsattel J.-P. G. Ma K. Louis E. D. (2011). Parallel fiber counts and parallel fiber integrated density are similar in essential tremor cases and controls. Acta Neuropathol. 121, 287–289. 10.1007/s00401-010-0776-9 21080179 PMC4209470

[B100] Lackey E. P. Moreira L. Norton A. Hemelt M. E. Osorno T. Nguyen T. M. (2024). Specialized connectivity of molecular layer interneuron subtypes leads to disinhibition and synchronous inhibition of cerebellar Purkinje cells. Neuron 112, 2333–2348.e6. 10.1016/j.neuron.2024.04.010 38692278 PMC11360088

[B101] Lanciego J. L. Luquin N. Obeso J. A. (2012). Functional neuroanatomy of the basal ganglia. Cold Spring Harb. Perspect. Med. 2, a009621–20. 10.1101/cshperspect.a009621 23071379 PMC3543080

[B102] Larsen K. (2021). The porcine cerebellin gene family. Gene 799, 145852. 10.1016/j.gene.2021.145852 34274480

[B103] Lee K. J. Kim H. Kim T. S. Park S. H. Rhyu I. J. (2004). Morphological analysis of spine shapes of Purkinje cell dendrites in the rat cerebellum using high-voltage electron microscopy. Neurosci. Lett. 359, 21–24. 10.1016/j.neulet.2004.01.071 15050702

[B104] Lee K. J. Kim H. Rhyu I. J. (2005). The roles of dendritic spine shapes in Purkinje cells. Cerebellum 4, 97–104. 10.1080/14734220510007842 16035191

[B105] Lee K. P. Choi S. Hong J. H. Ahuja M. Graham S. Ma R. (2014). Molecular determinants mediating gating of Transient Receptor Potential Canonical (TRPC) channels by stromal interaction molecule 1 (STIM1). J. Biol. Chem. 289, 6372–6382. 10.1074/jbc.M113.546556 24464579 PMC3945304

[B106] Lev-Ram V. Wong S. T. Storm D. R. Tsien R. Y. (2002). A new form of cerebellar long-term potentiation is postsynaptic and depends on nitric oxide but not cAMP. Proc. Natl. Acad. Sci. U. S. A. 99, 8389–8393. 10.1073/pnas.122206399 12048250 PMC123077

[B107] Lewis J. E. Maler L. (2002). Dynamics of electrosensory feedback: short-term plasticity and inhibition in a parallel fiber pathway. J. Neurophysiol. 88, 1695–1706. 10.1152/jn.2002.88.4.1695 12364499

[B108] Li B. Z. Sumera A. Booker S. A. McCullagh E. A. (2023). Current best practices for analysis of dendritic spine morphology and number in neurodevelopmental disorder research. ACS Chem. Neurosci. 14, 1561–1572. 10.1021/acschemneuro.3c00062 37070364 PMC10161226

[B109] Lippman Bell J. J. Lordkipanidze T. Cobb N. Dunaevsky A. (2010). Bergmann glial ensheathment of dendritic spines regulates synapse number without affecting spine motility. Neuron Glia Biol. 6, 193–200. 10.1017/S1740925X10000165 21044397 PMC3244562

[B110] Lisman J. (1989). A mechanism for the Hebb and the anti-Hebb processes underlying learning and memory. Proc. Natl. Acad. Sci. U. S. A. 86, 9574–9578. 10.1073/pnas.86.23.9574 2556718 PMC298540

[B111] Liu Y. Formisano L. Savtchouk I. Takayasu Y. Szabo G. Zukin R. S. (2010). A single fear-inducing stimulus induces a transcription-dependent switch in synaptic AMPAR phenotype. Nat. Neurosci. 13, 223–231. 10.1038/nn.2474 20037575 PMC3140064

[B112] Liu C. J. Ammon W. Siless V. Fogarty M. Wang R. Atzeni A. (2021). Quantification of volumetric morphometry and optical property in the cortex of human cerebellum at micrometer resolution. Neuroimage 244, 118627. 10.1016/j.neuroimage.2021.118627 34607020 PMC8603939

[B113] Liu Z. Jiang M. Liakath-Ali K. Sclip A. Ko J. Zhang R. S. (2022). Deletion of Calsyntenin-3, an atypical cadherin, suppresses inhibitory synapses but increases excitatory parallel-fiber synapses in cerebellum. Elife 11, e70664–35. 10.7554/eLife.70664 35420982 PMC9064300

[B114] Llano I. Dreessen J. Kano M. Konnerth A. (1991). Intradendritic release of calcium induced by glutamate in cerebellar Purkinje cells. Neuron 7, 577–583. 10.1016/0896-6273(91)90370-f 1681831

[B115] London M. Schreibman A. Haäusser M. Larkum M. E. Segev I. (2002). The information efficacy of a synapse. Nat. Neurosci. 5, 332–340. 10.1038/nn826 11896396

[B116] Loschky S. S. Spano G. M. Marshall W. Schroeder A. Nemec K. M. Schiereck S. S. (2022). Ultrastructural effects of sleep and wake on the parallel fiber synapses of the cerebellum. Elife 11, e84199–26. 10.7554/ELIFE.84199 36576248 PMC9797193

[B117] Louis E. D. Lee M. Babij R. Ma K. Cortés E. Vonsattel J. P. G. (2014). Reduced Purkinje cell dendritic arborization and loss of dendritic spines in essential tremor. Brain 137, 3142–3148. 10.1093/brain/awu314 25367027 PMC4240305

[B118] Lu H. Esquivel A. V. Bower J. M. (2009). 3D electron microscopic reconstruction of segments of rat cerebellar Purkinje cell dendrites receiving ascending and parallel fiber granule cell synaptic inputs. J. Comp. Neurol. 514, 583–594. 10.1002/cne.22041 19363797

[B119] Luján R. Aguado C. Ciruela F. Arus X. M. Martín-Belmonte A. Alfaro-Ruiz R. (2018a). SK2 channels associate with mGlu1α receptors and CaV2.1 channels in purkinje cells. Front. Cell. Neurosci. 12, 311–316. 10.3389/fncel.2018.00311 30283304 PMC6156379

[B120] Luján R. Aguado C. Ciruela F. Cózar J. Kleindienst D. de la Ossa L. (2018b). Differential association of GABAB receptors with their effector ion channels in Purkinje cells. Brain Struct. Funct. 223, 1565–1587. 10.1007/s00429-017-1568-y 29177691 PMC5869904

[B121] Magnus G. Xing J. Zhang Y. Han V. Z. (2023). Diversity of cellular physiology and morphology of Purkinje cells in the adult zebrafish cerebellum. J. Comp. Neurol. 531, 461–485. 10.1002/cne.25435 36453181

[B122] Mao H. Mediavilla T. Estévez-Silva H. Marcellino D. Sultan F. (2022). Increase of vesicular glutamate transporter 2 co-expression in the deep cerebellar nuclei related to skilled reach learning. Brain Res. 1782, 1–8. 10.1016/j.brainres.2022.147842 35192848

[B123] Mapelli L. Gagliano G. Soda T. Laforenza U. Moccia F. D’Angelo E. U. (2017). Granular layer neurons control cerebellar neurovascular coupling through an NMDA receptor/NO-dependent system. J. Neurosci. 37, 1340–1351. 10.1523/JNEUROSCI.2025-16.2016 28039371 PMC6596861

[B124] Marcaggi P. (2015). Cerebellar endocannabinoids: retrograde signaling from purkinje cells. Cerebellum 14, 341–353. 10.1007/s12311-014-0629-5 25520276

[B125] Marr D. (1969). A theory of cerebellar cortex. J. Physiol. 202, 437–470. 10.1113/jphysiol.1969.sp008820 5784296 PMC1351491

[B126] Martone M. E. Zhang Y. Simpliciano V. M. Carragher B. O. Ellisman M. H. (1993). Three-dimensional visualization of the smooth endoplasmic reticulum in purkinje cell dendrites. J. Neurosci. 13, 4636–4646. 10.1523/jneurosci.13-11-04636.1993 8229189 PMC6576343

[B127] Martone M. E. Pollock J. A. Jones Y. Z. Ellisman M. H. (1996). Ultrastructural localization of dendritic messenger RNA in adult rat hippocampus. J. Neurosci. 16, 7437–7446. 10.1523/jneurosci.16-23-07437.1996 8922399 PMC6579092

[B128] Masoli S. Solinas S. D’Angelo E. (2015). Action potential processing in a detailed Purkinje cell model reveals a critical role for axonal compartmentalization. Front. Cell. Neurosci. 9, 47–22. 10.3389/fncel.2015.00047 25759640 PMC4338753

[B129] Masoli S. Sanchez-Ponce D. Vrieler N. Abu-Haya K. Lerner V. Shahar T. (2024). Human Purkinje cells outperform mouse Purkinje cells in dendritic complexity and computational capacity. Commun. Biol. 7, 5. 10.1038/s42003-023-05689-y 38168772 PMC10761885

[B130] Mavroudis I. A. Petrides F. Manani M. Chatzinikolaou F. Ciobică A. S. Pădurariu M. (2017). Purkinje cells pathology in schizophrenia. A morphometric approach. Rom. J. Morphol. Embryol. 58, 419–424. 28730225

[B131] Mavroudis I. Petridis F. Kazis D. Njau S. N. Costa V. Baloyannis S. J. (2019). Purkinje cells pathology in Alzheimer’s disease. Am. J. Alzheimers. Dis. Other Demen. 34, 439–449. 10.1177/1533317519859200 31256608 PMC10653362

[B132] Mavroudis I. Kazis D. Petridis F. Chatzikonstantinou S. Karantali E. Njau S. (2021). Morphological and morphometric changes in the Purkinje cells of patients with essential tremor. Exp. Ther. Med. 23, 167–168. 10.3892/etm.2021.11090 35069848 PMC8753961

[B133] Miguez-Cabello F. Wang X.-T. Yan Y. Brake N. Alexander R. P. D. Perozzo A. M. (2025). GluA2-containing AMPA receptors form a continuum of Ca2+-permeable channels. Nature 641, 537–544. 10.1038/s41586-025-08736-2 40108453

[B134] Mitsumura K. Hosoi N. Furuya N. Hirai H. (2011). Disruption of metabotropic glutamate receptor signalling is a major defect at cerebellar parallel fibre-Purkinje cell synapses in staggerer mutant mice. J. Physiol. 589, 3191–3209. 10.1113/jphysiol.2011.207563 21558162 PMC3145934

[B135] Moccia F. Zuccolo E. Soda T. Tanzi F. Guerra G. Mapelli L. (2015). Stim and Orai proteins in neuronal Ca(2+) signaling and excitability. Front. Cell. Neurosci. 9, 153. 10.3389/fncel.2015.00153 25964739 PMC4408853

[B136] Mohrmann L. Seebach J. Missler M. Rohlmann A. (2024). Distinct alterations in dendritic spine morphology in the absence of β-neurexins. Int. J. Mol. Sci. 25, 1285. 10.3390/ijms25021285 38279285 PMC10817056

[B137] Mokhtar D. M. (2020). Patterns of organization of cerebellum and spinal cord of the red-tail shark (*Epalzeorhynchos bicolor*): histological, morphometrical, and immunohistochemical studies. Microsc. Microanal. 26, 1255–1263. 10.1017/S1431927620024563 33050970

[B138] Morizawa Y. M. Matsumoto M. Nakashima Y. Endo N. Aida T. Ishikane H. (2022). Synaptic pruning through glial synapse engulfment upon motor learning. Nat. Neurosci. 25, 1458–1469. 10.1038/s41593-022-01184-5 36319770

[B139] Morton S. M. Bastian A. J. (2004). Cerebellar control of balance and locomotion. Neuroscientist 10, 247–259. 10.1177/1073858404263517 15155063

[B140] Mugnaini E. (1983). The length of cerebellar parallel fibers in chicken and rhesus monkey. J. Comp. Neurol. 220, 7–15. 10.1002/cne.902200103 6643718

[B141] Mundel P. Reiser J. Borja A. Z. M. Pavenstädt H. Davidson G. R. Kriz W. (1997). Rearrangements of the cytoskeleton and cell contacts induce process formation during differentiation of conditionally immortalized mouse podocyte cell lines. Exp. Cell Res. 236, 248–258. 10.1006/excr.1997.3739 9344605

[B142] Mut-Arbona P. Sperlágh B. (2023). P2 receptor-mediated signaling in the physiological and pathological brain: from development to aging and disease. Neuropharmacology 233, 109541. 10.1016/j.neuropharm.2023.109541 37062423

[B143] Nakayama H. Miyazaki T. Abe M. Yamazaki M. Kawamura Y. Choo M. (2024). Direct and indirect pathways for heterosynaptic interaction underlying developmental synapse elimination in the mouse cerebellum. Commun. Biol. 7, 806–813. 10.1038/s42003-024-06447-4 38961250 PMC11222442

[B144] Napper R. M. A. Harvey R. J. (1988). Quantitative study of the Purkinje cell dendritic spines in the rat cerebellum. J. Comp. Neurol. 274, 158–167. 10.1002/cne.902740203 3209739

[B145] Naur P. Hansen K. B. Kristensen A. S. Dravid S. M. Pickering D. S. Olsen L. (2007). Ionotropic glutamate-like receptor delta2 binds D-serine and glycine. Proc. Natl. Acad. Sci. U. S. A. 104, 14116–14121. 10.1073/pnas.0703718104 17715062 PMC1955790

[B146] Nedelescu H. Abdelhack M. (2013). Comparative morphology of dendritic arbors in populations of purkinje cells in mouse sulcus and apex. Neural Plast. 2013, 948587. 10.1155/2013/948587 24312734 PMC3839124

[B147] Nedelescu H. Abdelhack M. Pritchard A. T. (2018). Regional differences in Purkinje cell morphology in the cerebellar vermis of male mice. J. Neurosci. Res. 96, 1476–1489. 10.1002/jnr.24206 29319237

[B148] Negri S. Faris P. Pellavio G. Botta L. Orgiu M. Forcaia G. (2020). Group 1 metabotropic glutamate receptors trigger glutamate-induced intracellular Ca2+ signals and nitric oxide release in human brain microvascular endothelial cells. Cell. Mol. Life Sci. 77, 2235–2253. 10.1007/s00018-019-03284-1 31473770 PMC11104941

[B149] Nguyen T. M. Thomas L. A. Rhoades J. L. Ricchi I. Yuan X. C. Sheridan A. (2023). Structured cerebellar connectivity supports resilient pattern separation. Nature 613, 543–549. 10.1038/s41586-022-05471-w 36418404 PMC10324966

[B150] Nimchinsky E. A. Sabatini B. L. Svoboda K. (2002). Structure and function of dendritic spines. Annu. Rev. Physiol. 64, 313–353. 10.1146/annurev.physiol.64.081501.160008 11826272

[B151] Nishiyama H. Linden D. J. (2004). Differential maturation of climbing fiber innervation in cerebellar vermis. J. Neurosci. 24, 3926–3932. 10.1523/JNEUROSCI.5610-03.2004 15102908 PMC6729416

[B152] Nishiyama J. Yasuda R. (2015). Biochemical computation for spine structural plasticity. Neuron 87, 63–75. 10.1016/j.neuron.2015.05.043 26139370 PMC4722820

[B153] Nitta A. Yamasaki M. Miyazaki T. Konno K. Yoshimura H. Watanabe M. (2025). Molecular and anatomical strengthening of “winner” climbing fiber synapses in developing mouse purkinje cells. J. Neurosci. 45, e2156242025. 10.1523/JNEUROSCI.2156-24.2025 40015986 PMC11984076

[B154] Nomura S. Yamasaki M. Miyazaki T. Konno K. Watanabe M. (2025). Preferential localization of STIM1 to dendritic subsurface ER structures in mouse purkinje cells. J. Neurosci. 45, e1829242025. 10.1523/JNEUROSCI.1829-24.2025 40086872 PMC12005364

[B155] Okamoto K. I. Narayanan R. Lee S. H. Murata K. Hayashi Y. (2007). The role of CaMKII as an F-actin-bundling protein crucial for maintenance of dendritic spine structure. Proc. Natl. Acad. Sci. U. S. A. 104, 6418–6423. 10.1073/pnas.0701656104 17404223 PMC1851051

[B156] Okubo Y. Suzuki J. Kanemaru K. Nakamura N. Shibata T. Iino M. (2015). Visualization of Ca2+ filling mechanisms upon synaptic inputs in the endoplasmic reticulum of cerebellar purkinje cells. J. Neurosci. 35, 15837–15846. 10.1523/JNEUROSCI.3487-15.2015 26631466 PMC6605451

[B157] Osorno T. Rudolph S. Nguyen T. Kozareva V. Nadaf N. M. Norton A. (2022). Candelabrum cells are ubiquitous cerebellar cortex interneurons with specialized circuit properties. Nat. Neurosci. 25, 702–713. 10.1038/s41593-022-01057-x 35578131 PMC9548381

[B158] Otsu Y. Marcaggi P. Feltz A. Isope P. Kollo M. Nusser Z. (2014). Activity-dependent gating of calcium spikes by A-type K+ channels controls climbing fiber signaling in purkinje cell dendrites. Neuron 84, 137–151. 10.1016/j.neuron.2014.08.035 25220810 PMC4183427

[B159] O’Brien J. Unwin N. (2006). Organization of spines on the dendrites of Purkinje cells. Proc. Natl. Acad. Sci. U. S. A. 103, 1575–1580. 10.1073/pnas.0507884103 16423897 PMC1360541

[B160] Palay S. L. Chan-Palay V. (1974a). Cerebellar cortex. Berlin, Heidelberg: Springer Berlin Heidelberg. 10.1007/978-3-642-65581-4

[B161] Palay S. L. Chan-Palay V. (1974b). “Granule cells,” in Cerebellar cortex: cytology and organization (Berlin, Heidelberg: Springer Berlin Heidelberg), 63–99. 10.1007/978-3-642-65581-4_3

[B162] Pali E. Masoli S. Di Domenico D. Sorbo T. Prestori F. D’Angelo E. (2025). Coincidence detection between apical and basal dendrites drives STDP in cerebellar Golgi cells. Commun. Biol. 8, 731–16. 10.1038/s42003-025-08153-1 40350534 PMC12066733

[B163] Palkovits M. Magyar P. Szentágothai J. (1971). Quantitative histological analysis of the cerebellar cortex in the cat. 3. Structural organization of the molecular layer. Brain Res. 34, 1–18. 10.1016/0006-8993(71)90347-7 5124919

[B164] Parajuli L. K. Urakubo H. Takahashi-Nakazato A. Ogelman R. Iwasaki H. Koike M. (2020). Geometry and the organizational principle of spine synapses along a dendrite. eNeuro 7, 0248–20.2020. 10.1523/ENEURO.0248-20.2020 33109633 PMC7772515

[B165] Park D. Bae S. Yoon T. H. Ko J. (2018). Molecular mechanisms of synaptic specificity: spotlight on hippocampal and cerebellar synapse organizers. Mol. Cells 41, 373–380. 10.14348/molcells.2018.0081 29665671 PMC5974614

[B166] Park C. Gim J. Bahn S. Kim G. H. Im Y. Lee S.-H. (2023). A cerebellar disinhibitory circuit supports synaptic plasticity. Bioarxiv. 10.1101/2023.09.15.557147

[B167] Paul M. A. Sigoillot S. M. Marti L. Urra Quiroz F. J. Delagrange M. Cheung H. W. (2024). Stepwise molecular specification of excitatory synapse diversity onto cerebellar Purkinje cells. Nat. Neurosci. 28, 308–319. 10.1038/s41593-024-01826-w 39658623

[B168] Pchitskaya E. Bezprozvanny I. (2020). Dendritic spines shape analysis—classification or clusterization? Perspective. Front. Synaptic Neurosci. 12, 31. 10.3389/fnsyn.2020.00031 33117142 PMC7561369

[B169] Peter S. Ten Brinke M. M. Stedehouder J. Reinelt C. M. Wu B. Zhou H. (2016). Dysfunctional cerebellar Purkinje cells contribute to autism-like behaviour in Shank2-deficient mice. Nat. Commun. 7, 12627. 10.1038/ncomms12627 27581745 PMC5025785

[B170] Piochon C. Irinopoulou T. Brusciano D. Bailly Y. Mariani J. Levenes C. (2007). NMDA receptor contribution to the climbing fiber response in the adult mouse Purkinje cell. J. Neurosci. 27, 10797–10809. 10.1523/JNEUROSCI.2422-07.2007 17913913 PMC6672834

[B171] Piochon C. Levenes C. Ohtsuki G. Hansel C. (2010). Purkinje cell NMDA receptors assume a key role in synaptic gain control in the mature cerebellum. J. Neurosci. 30, 15330–15335. 10.1523/JNEUROSCI.4344-10.2010 21068337 PMC2990192

[B172] Piochon C. Kruskal P. Maclean J. Hansel C. (2012). Non-Hebbian spike-timing-dependent plasticity in cerebellar circuits. Front. Neural Circuits 6, 124. 10.3389/fncir.2012.00124 23335888 PMC3542521

[B173] Piochon C. Kloth A. D. Grasselli G. Titley H. K. Nakayama H. Hashimoto K. (2014). Cerebellar plasticity and motor learning deficits in a copy-number variation mouse model of autism. Nat. Commun. 5, 5586. 10.1038/ncomms6586 25418414 PMC4243533

[B174] Pugh J. R. Raman I. M. (2005). GABAA receptor kinetics in the cerebellar nuclei: evidence for detection of transmitter from distant release sites. Biophys. J. 88, 1740–1754. 10.1529/biophysj.104.055814 15626699 PMC1305230

[B175] Purkinje jan E. (1837). Neueste Untersuchungen aus der Nerven-und Hirnanatomie. Amtlicher Ber. über Versamml. Ges. Dtsch. Naturforscher Aerzte, 177–180.

[B176] Raghu P. Joseph A. Krishnan H. Singh P. Saha S. (2019). Phosphoinositides: regulators of nervous system function in health and disease. Front. Mol. Neurosci. 12, 208. 10.3389/fnmol.2019.00208 31507376 PMC6716428

[B177] Ramón y Cajal S. (1888). Estructura de los centros nerviosos de las aves. Rev. Trimest. Histol. Norm. Patològica 1, 1–10.

[B178] Rapp M. Segev I. Yarom Y. (1994). Physiology, morphology and detailed passive models of Guinea-pig cerebellar Purkinje cells. J. Physiol. 474, 101–118. 10.1113/jphysiol.1994.sp020006 8014888 PMC1160299

[B179] Renzi F. Cull-Candy S. G. (2007). Climbing-fibre activation of NMDA receptors in Purkinje cells of adult mice. J. Physiol. 585, 91–101. 10.1113/jphysiol.2007.141531 17901118 PMC2327252

[B180] Rieke F. (1999). Spikes: exploring the neural code. Cambridge, Mass: MIT.

[B181] Risher W. C. Ustunkaya T. Alvarado J. S. Eroglu C. (2014). Rapid golgi analysis method for efficient and unbiased classification of dendritic spines. PLoS One 9, e107591. 10.1371/journal.pone.0107591 25208214 PMC4160288

[B182] Roberts P. D. Leen T. K. (2010). Anti-Hebbian spike-timing-dependent plasticity and adaptive sensory processing. Front. Comput. Neurosci. 4, 156–11. 10.3389/fncom.2010.00156 21228915 PMC3018773

[B183] Roesler M. K. Lombino F. L. Freitag S. Schweizer M. Hermans-Borgmeyer I. Schwarz J. R. (2019). Myosin XVI regulates actin cytoskeleton dynamics in dendritic spines of purkinje cells and affects presynaptic organization. Front. Cell. Neurosci. 13, 330. 10.3389/fncel.2019.00330 31474830 PMC6705222

[B184] Roth A. Hausser M. (2001). Compartmental models of rat cerebellar Purkinje cells based on simultaneous somatic and dendritic patch-clamp recordings. J. Physiol. 535, 445–472. 10.1111/j.1469-7793.2001.00445.x 11533136 PMC2278793

[B185] Rubio M. E. Soto F. (2001). Distinct localization of P2X receptors at excitatory postsynaptic specializations. J. Neurosci. 21, 641–653. 10.1523/jneurosci.21-02-00641.2001 11160443 PMC6763822

[B186] Runge K. Cardoso C. de Chevigny A. (2020). Dendritic spine plasticity: function and mechanisms. Front. Synaptic Neurosci. 12, 36. 10.3389/fnsyn.2020.00036 32982715 PMC7484486

[B187] Ryu K. Yokoyama M. Yamashita M. Hirano T. (2012). Induction of excitatory and inhibitory presynaptic differentiation by GluD1. Biochem. Biophys. Res. Commun. 417, 157–161. 10.1016/j.bbrc.2011.11.075 22138648

[B188] Ryu C. Jang D. C. Jung D. Kim Y. G. Shim H. G. Ryu H.-H. (2017). STIM1 regulates somatic Ca2+ signals and intrinsic firing properties of cerebellar purkinje neurons. J. Neurosci. 37, 8876–8894. 10.1523/JNEUROSCI.3973-16.2017 28821659 PMC6596796

[B189] Safo P. Cravatt B. Regehr W. (2006). Retrograde endocannabinoid signaling in the cerebellar cortex. Cerebellum 5, 134–145. 10.1080/14734220600791477 16818388

[B190] Santucci D. M. Raghavachari S. (2008). The effects of NR2 subunit-dependent NMDA receptor kinetics on synaptic transmission and CaMKII activation. PLoS Comput. Biol. 4, e1000208. 10.1371/journal.pcbi.1000208 18974824 PMC2563690

[B191] Schmahmann J. D. (2019). The cerebellum and cognition. Neurosci. Lett. 688, 62–75. 10.1016/j.neulet.2018.07.005 29997061

[B192] Schmidt H. (2019). Control of presynaptic parallel fiber efficacy by activity-dependent regulation of the number of occupied release sites. Front. Syst. Neurosci. 13, 30–36. 10.3389/fnsys.2019.00030 31379524 PMC6650762

[B193] Schonewille M. Girasole A. E. Rostaing P. Mailhes-Hamon C. Ayon A. Nelson A. B. (2021). NMDARs in granule cells contribute to parallel fiber-Purkinje cell synaptic plasticity and motor learning. Proc. Natl. Acad. Sci. U. S. A. 118, e2102635118–e2102635119. 10.1073/pnas.2102635118 34507990 PMC8449340

[B194] Sdrulla A. D. Linden D. J. (2007). Double dissociation between long-term depression and dendritic spine morphology in cerebellar Purkinje cells. Nat. Neurosci. 10, 546–548. 10.1038/nn1889 17435753

[B195] Seigneur E. Südhof T. C. (2017). Cerebellins are differentially expressed in selective subsets of neurons throughout the brain. J. Comp. Neurol. 525, 3286–3311. 10.1002/cne.24278 28714144 PMC5720827

[B196] Selimi F. Lohof A. M. Heitz S. Lalouette A. Jarvis C. I. Bailly Y. (2003). Lurcher GRID2-induced death and depolarization can Be dissociated in cerebellar Purkinje cells. Neuron 37, 813–819. 10.1016/S0896-6273(03)00093-X 12628171

[B197] Sereno M. I. Diedrichsen J. Tachrount M. Testa-Silva G. D Arceuil H. De Zeeuw C. (2020). The human cerebellum has almost 80% of the surface area of the neocortex. Proc. Natl. Acad. Sci. U. S. A. 117, 19538–19543. 10.1073/pnas.2002896117 32723827 PMC7431020

[B198] Sharp A. H. McPherson P. S. Dawson T. M. Aoki C. Campbell K. P. Snyder S. H. (1993). Differential immunohistochemical localization of inositol 1,4,5-trisphosphate- and ryanodine-sensitive Ca2+ release channels in rat brain. J. Neurosci. 13, 3051–3063. 10.1523/jneurosci.13-07-03051.1993 8392539 PMC6576698

[B199] Shouval H. Z. Bear M. F. Cooper L. N. (2002). A unified model of NMDA receptor-dependent bidirectional synaptic plasticity. Proc. Natl. Acad. Sci. U. S. A. 99, 10831–10836. 10.1073/pnas.152343099 12136127 PMC125058

[B200] Sigoillot S. M. Iyer K. Binda F. González-Calvo I. Talleur M. Vodjdani G. (2015). The secreted protein C1QL1 and its receptor Bai3 control the synaptic connectivity of excitatory inputs converging on cerebellar purkinje cells. Cell Rep. 10, 820–832. 10.1016/j.celrep.2015.01.034 25660030

[B201] Skibinska-Kijek A. Wisniewska M. B. Gruszczynska-Biegala J. Methner A. Kuznicki J. (2009). Immunolocalization of STIM1 in the mouse brain. Acta Neurobiol. Exp. (Wars). 69, 413–428. 10.55782/ane-2009-1753 20048759

[B202] Sotelo C. Dusart I. (2009). Intrinsic versus extrinsic determinants during the development of Purkinje cell dendrites. Neuroscience 162, 589–600. 10.1016/j.neuroscience.2008.12.035 19166910

[B203] Spacek J. Harris K. M. (1997). Three-dimensional organization of smooth endoplasmic reticulum in hippocampal CA1 dendrites and dendritic spines of the immature and mature rat. J. Neurosci. 17, 190–203. 10.1523/jneurosci.17-01-00190.1997 8987748 PMC6793680

[B204] Spanaki C. Sidiropoulou K. Petraki Z. Diskos K. Konstantoudaki X. Volitaki E. (2024). Glutamate-specific gene linked to human brain evolution enhances synaptic plasticity and cognitive processes. iScience 27, 108821. 10.1016/j.isci.2024.108821 38333701 PMC10850756

[B205] Stevenson M. E. Nazario A. S. Czyz A. M. Owen H. A. Swain R. A. (2021). Motor learning rapidly increases synaptogenesis and astrocytic structural plasticity in the rat cerebellum. Neurobiol. Learn. Mem. 177, 107339. 10.1016/j.nlm.2020.107339 33186744

[B206] Streng M. L. Carter R. E. Kottke B. W. Togneri K. Wasserman E. Rajendran V. (2025). Purkinje cell spatial correlation dynamics are key to cerebellar cortical contributions to behavior. J. Neurosci. 45, e1915242025. 10.1523/JNEUROSCI.1915-24.2025 40555517 PMC12311771

[B207] Südhof T. C. (2008). Neuroligins and neurexins link synaptic function to cognitive disease. Nature 455, 903–911. 10.1038/nature07456 18923512 PMC2673233

[B208] Südhof T. C. (2017). Synaptic neurexin complexes: a molecular code for the logic of neural circuits. Cell 171, 745–769. 10.1016/j.cell.2017.10.024 29100073 PMC5694349

[B209] Südhof T. C. (2023). Cerebellin–neurexin complexes instructing synapse properties. Curr. Opin. Neurobiol. 81, 102727. 10.1016/j.conb.2023.102727 37209532

[B210] Sugawara T. Hisatsune C. Le T. D. Hashikawa T. Hirono M. Hattori M. (2013). Type 1 inositol trisphosphate receptor regulates cerebellar circuits by maintaining the spine morphology of purkinje cells in adult mice. J. Neurosci. 33, 12186–12196. 10.1523/JNEUROSCI.0545-13.2013 23884927 PMC6618669

[B211] Sugawara T. Hisatsune C. Miyamoto H. Ogawa N. Mikoshiba K. (2017). Regulation of spinogenesis in mature Purkinje cells via mGluR/PKC-mediated phosphorylation of CaMKII. Proc. Natl. Acad. Sci. U. S. A. 114, E5256-E5265–E5265. 10.1073/pnas.1617270114 28607044 PMC5495224

[B212] Suk E. Lai K. Uesaka N. (2025). Reduced GABAergic inhibition and impaired synapse elimination by neuroligin-2 deletion from Purkinje cells of the developing cerebellum, 1–18. 10.3389/fncir.2025.1530141 PMC11949940 40160866

[B213] Szabó L. E. Marcello G. M. Süth M. Sótonyi P. Rácz B. (2021). Distribution of cortactin in cerebellar Purkinje cell spines. Sci. Rep. 11, 1375–11. 10.1038/s41598-020-80469-w 33446758 PMC7809465

[B214] Sziber Z. Torrents-Solé P. Kovacevic A. Kapfhammer J. P. (2025). Protein kinase C gamma regulates Purkinje cell dendritic spine development in a mouse model of spinocerebellar ataxia. Exp. Neurol. 393, 115377. 10.1016/j.expneurol.2025.115377 40675361

[B215] Tabata T. Kano M. (2006). GABAB receptor-mediated modulation of glutamate signaling in cerebellar Purkinje cells. Cerebellum 5, 127–133. 10.1080/14734220600788911 16818387

[B216] Takeo Y. H. Shuster S. A. Jiang L. Hu M. C. Luginbuhl D. J. Rülicke T. (2021). GluD2-and Cbln1-mediated competitive interactions shape the dendritic arbors of cerebellar Purkinje cells. Neuron 109, 629–644.e8. 10.1016/j.neuron.2020.11.028 33352118 PMC8833808

[B217] Tan L. Shi J. Moghadami S. Parasar B. Wright C. P. Seo Y. (2023). Lifelong restructuring of 3D genome architecture in cerebellar granule cells. Science 381, 1112–1119. 10.1126/science.adh3253 37676945 PMC11059189

[B218] Tao-Cheng J.-H. (2025). Ultrastructural characterization of peri-synaptic astrocytic processes around cerebellar Purkinje spines under resting and stimulated conditions. Mol. Brain 18, 28. 10.1186/s13041-025-01198-7 40165219 PMC11956224

[B219] Thomas R. E. Mudlaff F. Schweers K. Farmer W. T. Suvrathan A. (2024). Heterogeneity in slow synaptic transmission diversifies purkinje cell timing. J. Neurosci. 44, e0455242024. 10.1523/JNEUROSCI.0455-24.2024 39147589 PMC11391503

[B220] Tian W. Zhou J. Bartlett A. Zeng Q. Liu H. Castanon R. G. (2022). Epigenomic complexity of the human brain revealed by single-cell DNA methylomes and 3D genome structures. bioRxiv 11.30, 518285. 10.1101/2022.11.30.518285

[B221] Toledo A. Lang F. Doengi M. Morrison H. Stein V. Baader S. L. (2019). Merlin modulates process outgrowth and synaptogenesis in the cerebellum. Brain Struct. Funct. 224, 2121–2142. 10.1007/s00429-019-01897-7 31165301

[B222] Tolve M. Tutas J. Özer-Yildiz E. Klein I. Petzold A. Fritz V. J. (2025). The endocytic adaptor AP-2 maintains Purkinje cell function by balancing cerebellar parallel and climbing fiber synapses. Cell Rep. 44, 115256. 10.1016/j.celrep.2025.115256 39918958

[B223] Tomiyama M. Palacios J. M. Cortés R. Mengod G. (1999). Flip and flop variants of AMPA receptor subunits in the human cerebellum: implication for the selective vulnerability of Purkinje cells. Synapse 31, 163–167. 10.1002/(SICI)1098-2396(199902)31:2<163::AID-SYN10>3.0.CO;2-H 10024014

[B224] Tønnesen J. Katona G. Rózsa B. Nägerl U. V. (2014). Spine neck plasticity regulates compartmentalization of synapses. Nat. Neurosci. 17, 678–685. 10.1038/nn.3682 24657968

[B225] Uemura T. Lee S. J. Yasumura M. Takeuchi T. Yoshida T. Ra M. (2010). Trans-synaptic interaction of GluRdelta2 and Neurexin through Cbln1 mediates synapse formation in the cerebellum. Cell 141, 1068–1079. 10.1016/j.cell.2010.04.035 20537373

[B226] Uemura T. Suzuki-Kouyama E. Kawase S. Kurihara T. Yasumura M. Yoshida T. (2022). Neurexins play a crucial role in cerebellar granule cell survival by organizing autocrine machinery for neurotrophins. Cell Rep. 39, 110624. 10.1016/j.celrep.2022.110624 35385735

[B227] Uesaka N. Abe M. Konno K. Yamazaki M. Sakoori K. Watanabe T. (2018). Retrograde signaling from progranulin to Sort1 counteracts synapse elimination in the developing cerebellum. Neuron 97, 796–805. 10.1016/j.neuron.2018.01.018 29398357

[B228] Uriu Y. Kiyonaka S. Miki T. Yagi M. Akiyama S. Mori E. (2010). Rab3-interacting molecule γ isoforms lacking the rab3-binding domain induce long lasting currents but block neurotransmitter vesicle anchoring in voltage-dependent P/Q-type Ca2+ channels. J. Biol. Chem. 285, 21750–21767. 10.1074/jbc.M110.101311 20452978 PMC2898395

[B229] van der Heijden M. E. Sillitoe R. V. (2021). Interactions between purkinje cells and granule cells coordinate the development of functional cerebellar circuits. Neuroscience 462, 4–21. 10.1016/j.neuroscience.2020.06.010 32554107 PMC7736359

[B230] van der Heijden M. E. Lackey E. P. Perez R. Işleyen F. S. Brown A. M. Donofrio S. G. (2021). Maturation of Purkinje cell firing properties relies on neurogenesis of excitatory neurons. Elife 10, e68045–37. 10.7554/eLife.68045 34542409 PMC8452305

[B231] Van Overwalle F. (2024). Social and emotional learning in the cerebellum. Nat. Rev. Neurosci. 25, 776–791. 10.1038/s41583-024-00871-5 39433716

[B232] Vecellio M. Schwaller B. Meyer M. Hunziker W. Celio M. R. (2000). Alterations in Purkinje cell spines of calbindin D-28 k and parvalbumin knock-out mice. Eur. J. Neurosci. 12, 945–954. 10.1046/j.1460-9568.2000.00986.x 10762324

[B233] Verslegers M. Van Hove I. Dekeyster E. Gantois I. Hu T. T. D’Hooge R. (2014). MMP-2 mediates Purkinje cell morphogenesis and spine development in the mouse cerebellum. Brain Struct. Funct. 220, 1601–1617. 10.1007/s00429-014-0747-3 24652381

[B234] Vlachos A. Korkotian E. Schonfeld E. Copanaki E. Deller T. Segal M. (2009). Synaptopodin regulates plasticity of dendritic spines in hippocampal neurons. J. Neurosci. 29, 1017–1033. 10.1523/JNEUROSCI.5528-08.2009 19176811 PMC6665122

[B235] VoŽeh F. (2015). Jan Evangelista Purkyně and the cerebellum then and now. Physiol. Res. 64, S567–S584. 10.33549/physiolres.933231 26674295

[B236] Wagner W. Brenowitz S. D. Hammer J. A. (2011). Myosin-Va transports the endoplasmic reticulum into the dendritic spines of Purkinje neurons. Nat. Cell Biol. 13, 40–48. 10.1038/ncb2132 21151132 PMC3403743

[B237] Walter J. T. Dizon M.-J. Khodakhah K. (2009). The functional equivalence of ascending and parallel fiber inputs in cerebellar computation. J. Neurosci. 29, 8462–8473. 10.1523/JNEUROSCI.5718-08.2009 19571137 PMC4211897

[B238] Wang Y. Deng X. Mancarella S. Hendron E. Eguchi S. Soboloff J. (2010). The calcium store sensor, STIM1, reciprocally controls Orai and CaV1.2 channels. Science 330, 105–109. 10.1126/science.1191086 20929813 PMC3601900

[B239] Wang B. Lebel A. Mello A. M. D. (2025). Trends in Cognitive Sciences Ignoring the cerebellum is hindering progress in neuroscience. Trends Cogn. Sci. xx, 1–13. 10.1016/j.tics.2025.01.004 39934082

[B240] Wilms C. D. Häusser M. (2015). Reading out a spatiotemporal population code by imaging neighbouring parallel fibre axons *in vivo* . Nat. Commun. 6, 6464. 10.1038/ncomms7464 25751648 PMC4366501

[B241] Yamashita A. Makita K. Kuroiwa T. Tanaka K. (2006). Glutamate transporters GLAST and EAAT4 regulate postischemic Purkinje cell death: an *in vivo* study using a cardiac arrest model in mice lacking GLAST or EAAT4. Neurosci. Res. 55, 264–270. 10.1016/j.neures.2006.03.007 16647773

[B242] Yoast R. E. Emrich S. M. Zhang X. Xin P. Johnson M. T. Fike A. J. (2020). The native ORAI channel trio underlies the diversity of Ca2+ signaling events. Nat. Commun. 11, 2444. 10.1038/s41467-020-16232-6 32415068 PMC7229178

[B243] Yuste R. (2015). The discovery of dendritic spines by Cajal. Front. Neuroanat. 9, 18–6. 10.3389/fnana.2015.00018 25954162 PMC4404913

[B244] Yuste R. Bonhoeffer T. (2004). Genesis of dendritic spines: insights from ultrastructural and imaging studies. Nat. Rev. Neurosci. 5, 24–34. 10.1038/nrn1300 14708001

[B245] Yuzaki M. Aricescu A. R. (2017). A GluD coming-of-age story. Trends Neurosci. 40, 138–150. 10.1016/j.tins.2016.12.004 28110935 PMC5553105

[B246] Zárský V. (2012). Jan Evangelista Purkyně/Purkinje (1787-1869) and the establishment of cellular physiology--Wrocław/Breslau as a central European cradle for a new science. Protoplasma 249, 1173–1179. 10.1007/s00709-012-0407-5 22543689

[B247] Zeng W. Yuan J. P. Kim M. S. Choi Y. J. Huang G. N. Worley P. F. (2008). STIM1 gates TRPC channels, but not Orai1, by electrostatic interaction. Mol. Cell 32, 439–448. 10.1016/j.molcel.2008.09.020 18995841 PMC2586614

[B248] Zhang B. Chen L. Y. Liu X. Maxeiner S. Lee S.-J. Gokce O. (2015). Neuroligins sculpt cerebellar purkinje-cell circuits by differential control of distinct classes of synapses. Neuron 87, 781–796. 10.1016/j.neuron.2015.07.020 26291161 PMC4545494

[B249] Zhang R. S. Liakath-Ali K. Südhof T. C. (2020). Latrophilin-2 and latrophilin-3 are redundantly essential for parallel-fiber synapse function in cerebellum. Elife 9, e54443–21. 10.7554/eLife.54443 32202499 PMC7089768

[B250] Zheng J. Yang Q. Makris N. Huang K. Liang J. Ye C. (2023). Three-Dimensional digital reconstruction of the cerebellar cortex: lobule thickness, surface area measurements, and layer architecture. Cerebellum 22, 249–260. 10.1007/s12311-022-01390-8 35286708 PMC9470778

[B251] Zhu J. Qiu W. Wei F. Zhang J. Yuan Y. Liu L. (2024). Toll-like receptor 4 deficiency in Purkinje neurons drives cerebellar ataxia by impairing the BK channel-mediated after-hyperpolarization and cytosolic calcium homeostasis. Cell Death Dis. 15, 594. 10.1038/s41419-024-06988-w 39147737 PMC11327311

